# Rest-Activity Rhythm Is Associated With Obesity Phenotypes: A Cross-Sectional Analysis

**DOI:** 10.3389/fendo.2022.907360

**Published:** 2022-06-28

**Authors:** Jingen Li, Soumya Vungarala, Virend K. Somers, Junrui Di, Francisco Lopez-Jimenez, Naima Covassin

**Affiliations:** ^1^ Department of Cardiovascular Medicine, Dongzhimen Hospital, Beijing University of Chinese Medicine, Beijing, China; ^2^ Department of Cardiovascular Medicine, Mayo Clinic, Rochester, MN, United States; ^3^ Department of Biostatistics, Johns Hopkins University, Baltimore, MA, United States

**Keywords:** rest-activity rhythm, obesity, accelerometry, circadian rhythm, body fat

## Abstract

**Background:**

The prevalence of obesity continues to increase in spite of substantial efforts towards its prevention, posing a major threat to health globally. Circadian disruption has been associated with a wide range of preclinical and clinical disorders, including obesity. However, whether rest-activity rhythm (RAR), an expression of the endogenous circadian rhythm, is associated with excess adiposity is poorly understood. Here we aimed to assess the association of RAR with general and abdominal obesity.

**Methods:**

Non-institutionalized adults aged ≥20 years participating in the US National Health and Nutrition Examination Survey (NHANES) 2011-2014 who wore accelerometers for at least four 24-hour periods were included (N=7,838). Amplitude, mesor, acrophase and pseudo-F statistic of RAR were estimated using extended cosinor model, and interdaily stability (IS) and intradaily variability (IV) were computed by nonparametric methods. We tested the association between rest-activity rhythm and general obesity defined by body mass index and abdominal obesity by waist circumference. Waist-to-height ratio, sagittal abdominal diameter, and total and trunk fat percentages measured by imaging methods were also analyzed.

**Results:**

In multivariable analysis, low amplitude (magnitude of the rhythm), mesor (rhythm-corrected average activity level), pseudo-F statistic (robustness of the rhythm), IS (day-to-day rhythm stability), or high IV (rhythm fragmentation) were independently associated with higher likelihood of general or abdominal obesity (all *Ps*<.05). Consistently, RAR metrics were similarly associated with all adiposity measures (all *Ps*<.01). Delayed phase of RAR (later acrophase) was only significantly related to general and abdominal obesity in women.

**Conclusions:**

Aberrant RAR is independently associated with anthropometric and imaging measures of general and abdominal obesity. Longitudinal studies assessing whether RAR metrics can predict weight gain and incident obesity are warranted.

## Introduction

Obesity is a major risk factor for multiple noncommunicable diseases and may contribute to reduced survival (1, 2). Excess body weight also often leads to poor quality of life due to increased disability and social stigma (3). Notwithstanding substantial public health efforts, the prevalence of obesity continues to rise, having nearly tripled in adults since 1975 (4). Along with its societal and medical burden, there is pressing need to identify modifiable risk factors for obesity.

As circadian rhythms contribute to regulation of energy homeostasis and adipose tissue metabolism, circadian disruption has been identified as an independent risk factor for obesity (5), as evidenced in animal models (6), human with circadian gene variants (7–9), and shift workers (10–12). Mutations or knock out of key circadian genes (e.g., CLOCK, PER2) cause hyperphagia in mice, with animals consuming as much energy during the day as during the night, leading eventually to obesity (6, 13). In humans, several genetic circadian genes variants have been associated with greater risk of obesity, higher energy intake, lower energy expenditure and difficulty in achieving and maintaining weight loss (7–9). Night shift workers, who often experience chronic circadian misalignment, have 23% increased risk for obesity, and 35% for abdominal obesity (11). Rest-activity rhythm (RAR) is an evident manifestation of circadian rhythms and can be objectively assessed by accelerometers (14). Growing research links aberrant RAR patterns with poor health status, including greater risk of neurodegenerative disease (15–17), cardiometabolic disease (18) and mortality (19). Accelerometry-generated RAR metrics, including intradaily variability (IV, fragmentation of RAR) and interdaily stability (IS, day-to-day stability of RAR), have been associated with obesity in children, adolescents and in the elderly (20–23). However, the relationship of RAR with obesity among general adults remains unclear. Of note, obesity was defined solely by body mass index (BMI) in most of the above studies. While BMI is widely used to determine excess body weight, it has been criticized for its inability to differentiate between lean mass and fat mass, and to inform on fat mass distribution (24). The latter point is especially relevant as excess body fat in the abdominal region is a better predictor of morbidity and mortality than BMI (24). However, to the best of our knowledge, the association between RAR and abdominal obesity or body fat percentage has not been examined among general adults.

We aimed to investigate the association between accelerometry-generated RAR with anthropometric and imaging measures of general and abdominal obesity in a large sample of US adults from National Health and Nutrition Examination Surveys (NHANES) 2011-2012 and 2013-2014.

## Materials and Methods

### Study Design and Population

This cross-sectional analysis used data collected from NHANES, an ongoing, nationally representative, cross-sectional survey on nutrition and health of non-institutionalized US civilians. Accelerometry data were available for a total of 14,693 participants from NHANES 2011-2012 and 2013-2014 cycles. Days were regarded as valid when there was >80% wear time during the 24-hour period (25), with the device wear status ascertained by an open-source machine learning algorithm, which identified device wear status for each minute (26). Of the 14,693 participants, we excluded 2,152 participants with <4 days of valid accelerometry data (27), 4,266 aged <20 years, 75 pregnant, and 362 without BMI or waist circumference data. A total of 7,838 participants was thus included in the present analysis (**Supplementary Figure 1**). Characteristics of the population included in the current analysis were largely comparable to and thus representative of the non-pregnant US adult population (**Supplementary Table 1**). All participants provided written informed consent and the NHANES protocols were approved by the National Center for Health Statistics (NCHS) Ethics Review Board.

### Rest-Activity Rhythm Measures

Detailed information regarding generation of RAR metrics has been reported elsewhere (28). In brief, participants were instructed to wear an accelerometer (ActiGraph GT3X+, Pensacola, FL) on their non-dominant wrist for seven consecutive 24-hour periods. One-minute epochs of triaxial acceleration were summarized using Monitor-Independent Movement Summary (MIMS) units (29) and were used to compute RAR metrics.

**Figure 1:**
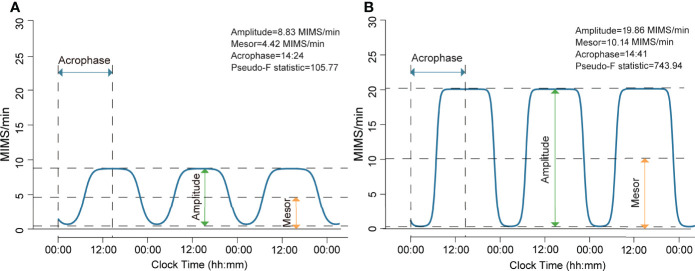
Rest-activity rhythm from participants with and without general obesity generated using extended cosinor model. **(A)** This participant had a BMI of 44.7 kg/m^2^ and was in the lowest quartiles of amplitude and mesor, indicating a lower magnitude of the rest-activity rhythm and a lower rhythm-adjusted average activity level. This participant exhibited a normal phase of rest-activity rhythm, as shown by the activity rhythm peaking at 14:24, and did not have distinct rest-activity periods as indicated by low rhythm robustness (pseudo-F statistic). **(B)** This participant had a BMI of 20.3 kg/m^2^ and was in the highest quartiles of amplitude and mesor, indicating a higher magnitude of the rest-activity rhythm and a higher rhythm-adjusted average activity level. This participant exhibited a normal phase of rest-activity rhythm, as shown by the activity rhythm peaking at 14:41, and had distinct rest-activity periods as indicated by the high value for rhythm robustness (pseudo-F statistic).

We excluded the first and the last days for each participant due to partial/incomplete data. Both the extended cosinor model (30–32) and the nonparametric method (33) were used to quantify RAR metrics. The extended cosinor model utilizes an antilogistic transformation to fit the activity data to a squared wavev (32), which has been shown to better depict human activity patterns than a regular cosinor shape (31). We analyzed the following RAR measures estimated from the extended cosinor: amplitude (MIMS/min), mesor (MIMS/min), acrophase (hh:mm), and pseudo-F statistic. Amplitude is calculated as the difference between the peak and trough of the fitted extended cosinor curve, and represents the magnitude or height of the RAR. Mesor represents the rhythm-adjusted mean activity level based on the fitted curve, with higher values indicating higher average activity levels. Acrophase is the clock time of peak activity of the fitted curve. Finally, pseudo-F statistic, is an adjusted measure of goodness-of-fit and represents robustness of the rhythm, with higher values suggesting greater rhythmicity. Examples of parametric RAR variables from two representative individuals with (**Figure 1A**) and without obesity (**Figure 1B**) are presented in **Figure 1**. The following non-parametric RAR metrics were calculated: 1) interdaily stability (IS; range of values 0 to 1), an index of stability of day-to-day rest-activity patterns, with greater values indicating more stable and consistent RAR across days; 2) intradaily variability (IV; range of values 0 to 2), an index reflecting fragmentation of the 24-hour RAR, with higher values indicating greater RAR disruption. Detailed definition of all RAR variables is also presented in **Supplementary Table 2**. All RAR measures were generated from 1-min epoch length. Histograms depicting data distribution of all RAR variables and scatter plots for bivariate correlations between each pair of RAR measures are provided as **Supplementary Figures 2, 3**, respectively.

### Anthropometric and Imaging Obesity Measures

Height (cm), weight (kg), waist circumference (cm), and sagittal abdominal diameter (SAD, cm) were all measured following standard procedures (**Supplementary Table 3**). BMI (kg/m^2^) was derived from height and weight and waist-to-height ratio (WHtR) was calculated as waist circumference divided by height. Total and trunk fat percentage (%) were quantified from whole-body dual-energy x-ray absorptiometry (DEXA) scans (Hologic, Inc., Bedford, MA) using software Apex 3.2 and were available in 4567 (58.3%) of the 7838 participants. General obesity was defined as BMI ≥30 kg/m^2^, while abdominal obesity was defined as waist circumference >102 cm for men and >88 cm for women (34).

### Covariate Assessment

Age, sex, race/ethnicity, education, family poverty income ratio, marital status, diet quality, self-reported general health status, alcohol consumption, smoking status, employment status, sleep duration, depression (35), analgesic or painkiller use, and diagnosed sleep disorders were obtained from in-home interviews using questionnaires. Details of these covariates are listed in **Supplementary Table 4**. Self-reported physical activity was collected using the Global Physical Activity Questionnaire (GPAQ) (36) and presented as metabolic equivalents (MET-minutes/week).

### Statistical Analysis

Categorical variables are reported as number (weighted percentage) and continuous variables as weighted means (standard errors [SE]). Comparisons of population characteristics across obesity groups were performed using the Rao-Scott χ^2^ test for categorical variables and ANOVA for continuous variables (37). RAR metrics were assessed as both continuous and categorical variables. Amplitude, mesor, pseudo-F statistics, IS, and IV were categorized into quartiles using the entire sample, while acrophase was categorized into three groups in accordance with previous studies (30, 31): phase advanced (1 SD less than mean, before 12:44), phase delayed (1 SD or greater than mean, 16:51 or after), and normal phase (mean ± 1SD, 12:44 to <16:51).

The association of RAR metrics, both categorical and continuous variables, with obesity was assessed using multivariable logistic regression adjusting for age, sex, race, education, marital status, employment status, poverty income ratio, diet quality, self-reported general health status, alcohol consumption, smoking status, and sleep duration. Odds ratios (ORs) with 95% confidence interval (CI) are reported for each RAR metric. The association of RAR metrics as continuous variables with BMI, waist circumference, SAD, total body fat percentage, trunk fat percentage, and WHtR was examined using multivariable linear regression adjusting for the covariates listed above, and standardized coefficients (β) are presented. Interaction effects and stratified analyses by sex and age (≥65 or <65years) were also performed. We also conducted several sensitivity analyses by further adjusting for potential confounders (i.e., self-reported physical activity levels, objective physical activity [mesor], depression, diagnosed sleep disorders and analgesic use) separately; by excluding those person-days with non-wear time; by excluding those who reached their peak activity level (acrophase) between 23:00 and 04:00. Additionally, to test for potential effects of different data length (number of days) on the association between nonparametric RAR metrics and obesity measures, we analyzed the association of IS and IV with obesity by including only participants for whom we had full 7-day data (n=6005). For all analyses, we took complex survey design factors into account as recommended. Accelerometry data were processed using the “ActCR” package in R (version 4.0.0) and statistical analyses were performed using SPSS 20.0 (IBM Corp) and R (version 4.0.0). A two-sided *P*-value of <.05 was considered statistically significant.

## Results

Participant characteristics and RAR metrics are showed in **Table 1**. The mean age of all participants was 48.66 (0.44) years, with 48.1% being men and 67.2% Non-Hispanic Whites. The mean number of valid wear days was 6.62 (0.01). Of the total 51,984 person-days, 49,129 (94.5%) person-days had 100% wear time and 1,347 (47.2%) of the 2,855 (5.5%) person-days with non-wear time had ≤2 hours of non-wear time (**Supplementary Figure 4**).

**Table 1:** Participant characteristics and RAR metrics according to general and abdominal obesity status.

Characteristics	Overall (n=7838)	General obesity		*P*	Abdominal obesity		*P*
		No (n=4867)	Yes (n=2971)		No (n= 3459)	Yes (n=4379)	
Men, n (%)	3830 (48.1)	2550 (49.8)	1280 (45.4)	.006	2205 (60.9)	1625 (38.7)	<.001
Age, year, mean (SE)	48.66 (0.44)	48.24 (0.52)	49.35 (0.50)	.06	44.74 (0.62)	51.56 (0.38)	<.001
Race, n (%)				<.001			<.001
Hispanic	1695 (14.4)	976 (13.3)	719 (16.3)		704 (14.9)	991 (14.1)	
NH-White	3197 (67.2)	2000 (68.3)	1197 (65.5)		1258 (64.0)	1939 (69.7)	
NH-Black	1820 (10.9)	953 (9.1)	867 (13.9)		711 (10.1)	1109 (11.6)	
NH-Asian	896 (4.6)	796 (6.6)	100 (1.4)		678 (8.3)	218 (2.0)	
Married/living with a partner, n (%)	4570 (63.1)	2891 (63.8)	1679 (62.0)	.23	2063 (63.4)	2507 (63.0)	.82
Ever attended college, n (%)	4393 (63.2)	2831 (65.4)	1562 (59.2)	<.001	2037 (66.1)	2356 (61.1)	<.001
Employed	4245 (60.7)	2716 (61.6)	1529 (59.3)	.12	2103 (66.5)	2142 (56.5)	<.001
Income poverty ratio<1, n (%)	1680 (15.7)	993 (15.2)	687 (16.5)	.131	693 (15.6)	987 (15.8)	.83
Smoking status, n (%)				.030			<.001
Never	4421 (55.7)	2742 (56.3)	1679 (54.7)		1980 (57.9)	2441 (54.1)	
Former	1859 (24.9)	1100 (23.4)	759 (27.2)		706 (20.5)	1153 (28.0)	
Current	1551 (19.5)	1021 (20.3)	530 (18.1)		769 (21.6)	782 (17.9)	
Alcohol consumption, n (%)	5366 (78.6)	3385 (80.6)	1981 (75.3)	<.001	2480 (83.2)	2886 (75.3)	<.001
Diet quality, n (%)				<.001			<.001
Excellent/very good	2363 (32.5)	852 (40.8)	323 (21.5)		1288 (40.5)	1075 (26.6)	
Good	3364 (42.6)	998 (41.5)	745 (45.0)		1489 (41.1)	1875 (43.6)	
Fair/poor	2107 (25.0)	467 (17.7)	622 (33.5)		682 (18.4)	1425 (29.8)	
Health status, n (%)				<.001			<.001
Excellent/very good	2698 (43.0)	562 (14.0)	143 (5.5)		1522 (55.7)	1176 (33.8)	
Good	2982 (39.5)	1772 (35.7)	1281 (45.6)		1176 (32.5)	1806 (44.5)	
Fair/poor	1713 (17.5)	758 (11.5)	761 (21.5)		531 (11.8)	1182 (21.6)	
Analgesic use	982 (12.3)	491 (10.1)	491 (16.0)	<.001	285 (8.2)	697 (15.3)	<.001
Depression	674 (8.2)	325 (6.5)	349 (11.0)	<.001	209 (6.2)	465 (9.7)	<.001
Diagnosed sleep disorders	767 (9.9)	279 (6.0)	488 (16.5)	<.001	163 (5.2)	604 (13.5)	<.001
Sleep duration, hrs, mean (SE)	6.91 (0.02)	6.98 (0.02)	6.80 (0.03)	<.001	6.97 (0.03)	6.87 (0.03)	.02
Self-reported PA, MET- minutes/week, mean (SE)	3253.65 (105.63)	3000.71 (148.70)	3407.11 (114.80)	.01	3993.02 (144.39)	2708.10 (114.00)	<.001
BMI, kg/m^2^, mean (SE)	29.08 (0.14)	25.00 (0.07)	35.81 (0.19)	<.001	24.06 (0.07)	32.79 (0.18)	<.001
Waist circumference, cm, mean (SE)	99.79 (0.30)	90.67 (0.27)	114.81 (0.40)	<.001	86.79 (0.21)	109.39 (0.30)	<.001
SAD, cm, mean (SE)	22.78 (0.09)	20.32 (0.08)	26.90 (0.10)	<.001	19.40 (0.06)	25.31 (0.10)	<.001
WHtR, mean (SE)	0.593 (0.002)	0.538 (0.002)	0.685 (0.003)	<.001	0.512 (0.001)	0.653 (0.002)	<.001
Trunk fat percentage (%), mean (SE)	32.19 (0.22)	28.53 (0.23)	38.31 (0.23)	<.001	26.00 (0.19)	37.79 (0.16)	<.001
Total fat percentage (%), mean (SE)	33.23 (0.21)	30.43 (0.23)	38.22 (0.23)	<.001	27.95 (0.19)	38.08 (0.16)	<.001
Amplitude, MIMS/min, mean (SE)	14.01 (0.10)	14.60 (0.17)	13.03 (0.13)	<.001	14.98 (0.19)	13.29 (0.10)	<.001
Mesor, MIMS/min, mean (SE)	8.16 (0.05)	8.44 (0.09)	7.70 (0.06)	<.001	8.66 (0.10)	7.79 (0.05)	<.001
Acrophase, n (%)				.37			.24
Advanced (<12:44)	710 (8.1)	428 (7.9)	254 (8.5)		315 (8.1)	367 (8.1)	
Normal (12:44-<16:51)	6455 (81.7)	3871 (82.4)	2368 (80.9)		2698 (81.0)	3541 (82.5)	
Delayed (≥16:51)	952 (10.2)	568 (9.7)	349 (10.6)		446 (10.9)	471 (9.4)	
Pseudo-F statistic, mean (SE)	240.56 (4.80)	251.28 (5.92)	222.90 (5.19)	<.001	252.93 (6.41)	231.43 (5.75)	.008
IS, mean (SE)	0.37 (0.002)	0.37 (0.002)	0.36 (0.002)	<.001	0.37 (0.003)	0.36 (0.002)	.006
IV, mean (SE)	0.43 (0.001)	0.42 (0.002)	0.45 (0.002)	<.001	0.42 (0.002)	0.44 (0.001)	<.001

All estimates accounted for complex survey design. Data are presented as number with weighted percentage (%) or weighted means with standard error (SE). BMI, body mass index; NH, non-Hispanic; IS, interdaily stability; IV, intradaily variability; PA, physical activity; SE, standard error.

Overall, 37.8% of participants exhibited general obesity, while abdominal obesity was present in 57.5% of the sample. Participants with general or abdominal obesity were more likely to be women and to have poor diet quality, depression, diagnosed sleep disorders, poor perceived health status, shorter sleep duration and lower self-reported physical activity level (**Table 1**).

### Association of RAR With Obesity Measures

**Figure 2:**
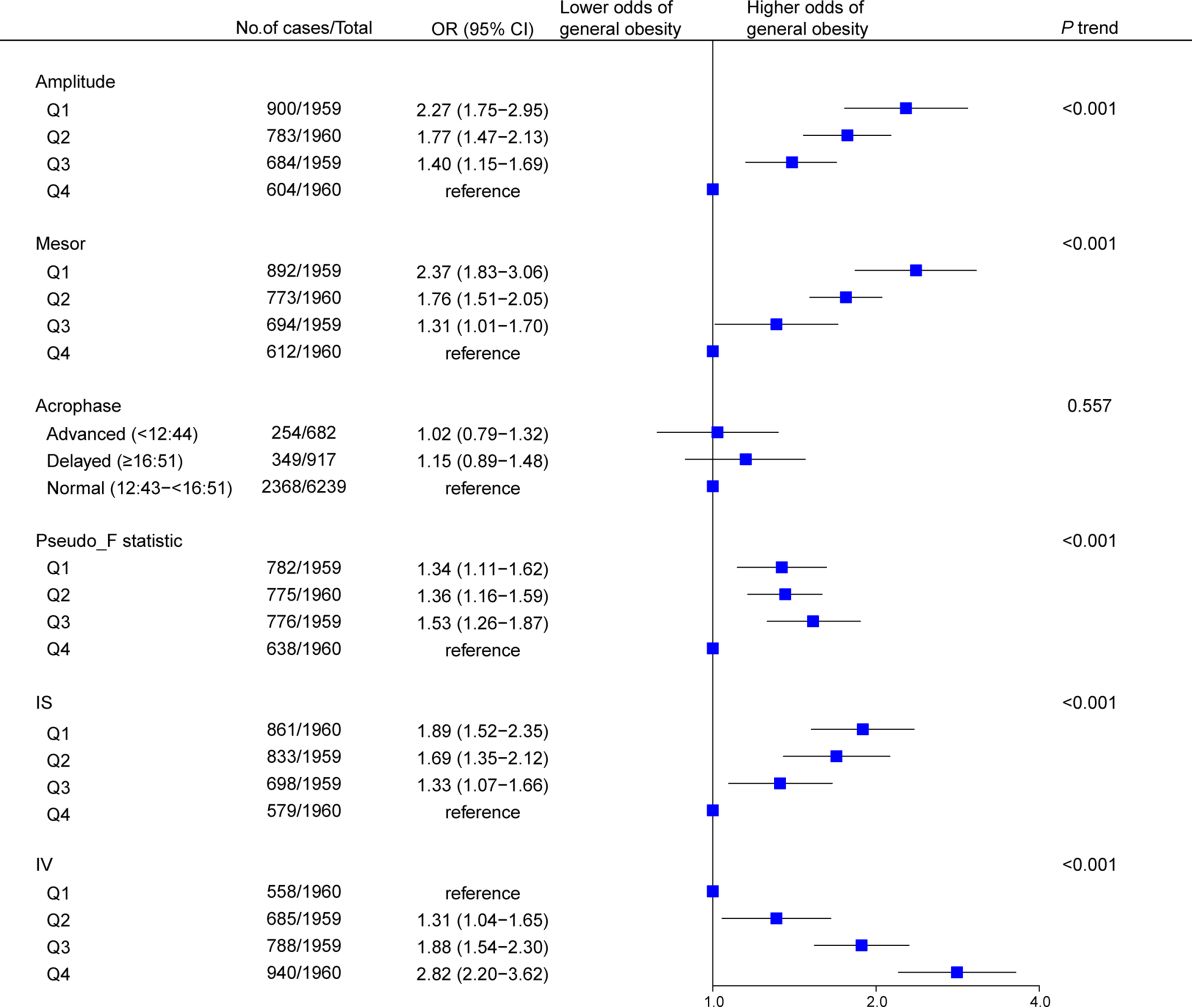
Association between rest-activity rhythm parameters and general obesity among US adults (n=7838). Odds ratios (datapoints) and 95% confidence interval (error bars) are presented. CI, confidence interval; IS, interdaily stability; IV, intradaily variability; OR, odds ratio.

**Figure 3:**
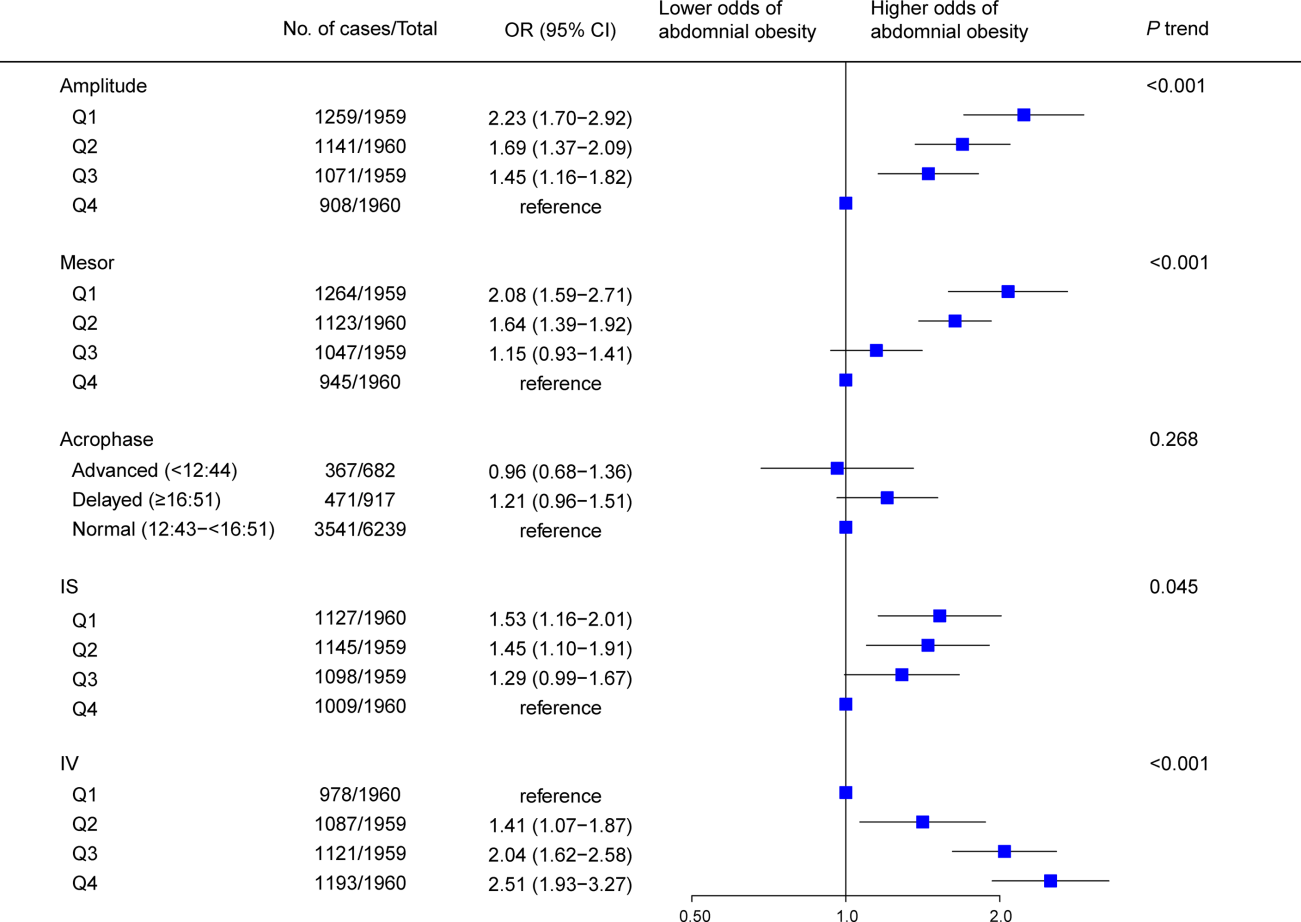
Association between rest-activity rhythm parameters and abdominal obesity among US adults (n=7838). Odds ratio (datapoints) and 95% confidence interval (error bars) are presented. CI, confidence interval; IS, interdaily stability; IV, intradaily variability; OR, odds ratio.

Results of multivariable analyses showed a graded relation between RAR and obesity, with progressively higher odds of both general (**Figure 2**) and abdominal (**Figure 3**) obesity across quartiles of RAR measures. Lower RAR magnitude (amplitude, Q1 vs Q4, OR [95% CI]: 2.27 [1.75-2.95] for general obesity, 2.23 [1.70-2.92] for abdominal obesity) and rhythm-corrected average activity level (mesor, OR [95% CI]: 2.37 [1.83-3.06] for general obesity, 2.08 [1.59-2.71] for abdominal obesity) were significantly associated with higher odds of general and abdominal obesity. Lower RAR stability (IS, Q1 vs Q4, OR [95% CI]: 1.89 [1.52-2.35] for general obesity, 1.53 [1.16-2.01] for abdominal obesity) was related with significantly higher odds of both obesity types, and lower RAR robustness (pseudo-F statistic, Q1 vs Q4, OR [95% CI]: 1.34 [1.11-1.62]) was associated with general obesity. Because we observed a significant pseudo-F statistic × sex interaction (*P* interaction <0.001), we analyzed the association of RAR robustness with abdominal obesity in women and men separately in the stratified analysis below. Lastly, RAR fragmentation (higher IV) was associated with greater odds of both general (Q4 vs. Q1, OR [95% CI], 2.82 [2.20-3.62] and abdominal obesity (OR [95% CI], 2.51 [1.93-3.27]) (**Figures 2**, **3**). Similar results were observed when these RAR variables were analyzed as standardized continuous variables (**Supplementary Table 5**).

Regarding adiposity measures, results of multivariable linear regression showed that BMI and waist circumference were inversely associated with RAR magnitude (amplitude), average activity level (mesor), robustness (pseudo-F statistic), and regularity (IS), but positively associated with RAR fragmentation (IV) (all *Ps<*.001; **Table 2** and **Supplementary Table 6**). Results were consistent when considering other adiposity measures, namely WHtR, SAD, total fat percentage and trunk fat percentage (all *Ps≤*.001, **Table 2**).

**Table 2:** Multivariable linear regression results of the association between RAR metrics and obesity measures.

RAR measures	BMI (n=7838)		Waist circumference (n=7838)		WHtR (n=7838)		SAD (n=7667)		Total body fat percentage (n=4382)		Trunk fat percentage (n=4567)	
	β	*P* value	β	*P* value	β	*P* value	β	*P* value	β	*P* value	β	*P* value
Amplitude	**-0.12**	<.001	**-0.13**	<.001	**-0.12**	<.001	**-0.13**	<.001	**-0.11**	<.001	**-0.11**	<.001
Mesor	**-0.10**	<.001	**-0.11**	<.001	**-0.13**	<.001	**-0.10**	<.001	**-0.09**	<.001	**-0.09**	<.001
Acrophase	0.01	.78	0.04	.82	0.01	.64	0.01	.50	0.02	.34	0.03	.24
Pseudo-F statistic	**-0.07**	<.001	**-0.07**	<.001	**-0.06**	<.001	**-0.06**	<.001	**-0.05**	.001	**-0.07**	<.001
IS	**-0.14**	<.001	**-0.14**	<.001	**-0.13**	<.001	**-0.13**	<.001	**-0.07**	<.001	**-0.08**	<.001
IV	**0.21**	<.001	**0.23**	<.001	**0.21**	<.001	**0.23**	<.001	**0.15**	<.001	**0.17**	<.001

β represents standardized coefficient. Bold values indicate significant associations (P values ≤.001). Adjusted variables included age, sex, race, education, marital status, employment status, poverty income ratio, diet quality, self-reported general health status, alcohol consumption, smoking status, and sleep duration. BMI, body mass index; IS, interdaily stability; IV, intradaily variability; SAD, sagittal abdominal diameter; WHtR, waist-to-height ratio.

### Sex- and Age-Stratified Analyses

Interaction effects between RAR metrics and sex were largely nonsignificant for both general and abdominal obesity, except for the association of pseudo-F statistic with abdominal obesity (**Supplementary Figures 5A**, **6A**). In stratified analysis pseudo-F statistic (RAR robustness) was significantly associated with abdominal obesity only in women (Q1 vs. Q4, OR [95%CI], 1.80 [1.31-2.46]; *P*_interaction_ <.001, **Supplementary Figure 6A**). Although there was no significant interaction between acrophase and sex for both general and abdominal obesity (**Supplementary Figures 5A** and **6A**), delayed RAR acrophase (later time of rhythm’s peak) was associated with significantly higher odds of both general (OR [95% CI]: 1.38 [1.02-1.87]) and abdominal (OR [95% CI]: 1.40, [1.04-1.88]) obesity in women but not in men. The relationship of RAR and either general or abdominal obesity did not differ significantly in adults aged ≥65 years vs those aged <65 years (all *Ps*_interaction_ >.05, **Supplementary Figures 5B**, **6B**).

### Additional Analysis

In sensitivity analysis, additional adjustment for mesor attenuated the observed associations between RAR measures and obesity, while additional adjustment for other factors (i.e., self-reported physical activity, depression, diagnosed sleep disorders and analgesic use) generated results similar to those obtained from the main analysis (**Supplementary Table 7**). Results of sensitivity analyses by excluding those who reached their peak activity level between 23:00 and 04:00 and by excluding the person-days with non-wear days were largely comparable to those from the main analysis (**Supplementary Figures 7, 8** and **Supplementary Table 8**). Lastly, analysis of the association of IS and IV with obesity measures including only participants with full 7-day data yielded again similar results (**Supplementary Tables 9 and 10**).

## Discussion

In this large, nationally representative sample of US adults, we found evidence of a graded association between RAR metrics and obesity measures ― participants with erratic (i.e., low IS or pseudo-F statistic values), fragmented (i.e., high IV values), or dampened RAR (i.e., low amplitude or mesor values) were more likely to have general or abdominal obesity compared to those exhibiting greater magnitude, robustness, or regularity of RAR, with odds being 2-3 times greater when comparing highest to lowest quartiles. Consistently, RAR magnitude (amplitude, mesor) and robustness/regularity (pseudo-F statistic, IS) indices were inversely while fragmentation index (IV) was positively associated with BMI, waist circumference and secondary measures of excess adiposity (i.e., WHtR, SAD, and total and trunk body fat percentage). Notably, the relationship between RAR and obesity appeared more robust in women than in men. Later timing of peak activity was also associated with higher odds of general and abdominal obesity only among women.

To the best of our knowledge, this is the first study to systematically assess the association of accelerometry-measured RAR metrics with excess body fat in the general population. Pooling accelerometry data from three different studies (N=578) to assess the relation between BMI and RAR magnitude, regularity, and timing, Cespedes Feliciano et al. (38) found only a significant, inverse association between BMI and RAR magnitude. This apparent discrepancy with our findings may result from the fact that, of the three original studies, the largest one with 329 participants only enrolled women, with BMI derived from self-reported height and weight and ranging between 21 and 39.9 kg/m^2^. As individuals tend to under-report their weight and over-report their height (39), BMI generated from self-reported measures may be inaccurate, leading to misclassifications and potentially biasing the association between RAR and obesity. Analysis of baseline data of a randomized trial of 97 patients with metabolic syndrome showed that, of all RAR metrics analyzed, only amplitude was inversely correlated with BMI in women, possibly because of the strict inclusion and exclusion criteria and the small sample size (40). Sohail et al. (23) assessed the relationship between RAR stability (IS) and metabolic syndrome in the very elderly and found that low IS was associated with increased risk of BMI-based general obesity. Of note, these previous studies (22, 23, 38, 40) evaluated RAR in relation to excess body fat only as expressed by BMI. Abdominal obesity, defined according to waist circumference or waist-to-hip ratio, is an indicator of visceral fat accumulation (41) and is a more powerful predictor of chronic diseases and premature death than BMI (42, 43). SAD and WHtR have also been reported as predictors of cardiovascular disease independent of BMI, and proposed as surrogate measures of visceral fat (42–44). In this study, we found that RAR metrics were also significantly associated with these measures, in agreement with prior findings from adolescents (20, 21).

Notably, the observed association between RAR and excess adiposity estimated by anthropometrics was confirmed by the significant correlation of RAR with total and trunk fat percentage quantified using DEXA, a method more accurate than anthropometry and comparable to the gold standard computed tomography in assessing body fat (45). Consistency of the associations between RAR and obesity risk across multiple measures of adiposity warrant reliability of our findings.

The association we observed between RAR and obesity is in accordance with previous studies that identified circadian disruption as a determinant of excess body fat accumulation (10, 46). A growing body of experimental data suggests that the association between circadian disruption and obesity can be attributed to dysregulation of energy homeostasis caused by altered rhythmicity of hormones, primarily melatonin, leptin, and glucocorticoids (46, 47).Later timing of food intake, which often results from circadian misalignment (48), has also been strongly linked to weight gain and obesity (49, 50), with potential mediating mechanisms including reduced resting and postprandial energy expenditure and increased insulin resistance (51, 52). On the other hand, because the fasting-feeding cycle contributes to synchronise peripheral circadian rhythms with the central clock, shifted mealtimes may also evoke or aggravate circadian disruption by uncoupling peripheral and central clocks (53). In addition, emerging evidence also implies derangements in gut microbiota, a critical factor for energy homeostasis, following circadian misalignment (54, 55).

In a previous study, we found that RAR patterns vary by age and sex (28). However, our age-stratified results showed that younger and older adults with disrupted RAR are at similar risk of having excess adiposity, implying that age was not an effect modifier of the association with obesity. Conversely, we found that the association between robustness of RAR (pseudo-F statistic) and abdominal obesity appeared to be stronger in women than men, and that delayed acrophase was associated with significantly greater risk of general and abdominal obesity only among women. These findings are in agreement with observational reports that female shift workers have greater risk of obesity or metabolic syndrome than men (56). An experimental study (57) found that circadian misalignment led to decreases in leptin, the satiety hormone, and increases in ghrelin, the hunger hormone, only in women, while men showed increases in leptin and no changes in ghrelin, suggesting that altered RAR may predispose to obesity in women by increasing energy intake signalling.

Although further, longitudinal studies confirming the relation between poor RAR and excess body weight are needed, our findings might have implications for obesity management and prevention in light of current evidence. As a recent study indicated that exercise in the morning might be more effective for weight loss than exercise in the afternoon (58), future interventions assessing whether improving magnitude, regularity and/or phase of RAR can provide protection against weight gain are warranted.

A major strength of our study is the application of objective measurements of both obesity and rest-activity patterns. By assessing multiple anthropometric and imaging indices of excess adiposity, together with parametric and non-parametric RAR metrics, we provided a comprehensive characterization of the relation between RAR and obesity. The present study also has limitations. First, because this is a cross-sectional investigation, no causal inferences can be drawn, although adjustment for numerous potential confounders in sensitivity analysis, including depression, diagnosed sleep disorders and analgesic use, overall did not alter the findings. Similar to the relation between RAR and neurodegenerative disorders (15), such as Alzheimer’s disease (17), the association between RAR and obesity could be bidirectional, as obesity could also affect rest-activity patterns through different mechanisms (59) In this regard, objectively measured physical activity partially explained the observed relations. Second, we cannot verify alignment of RAR measures with conventional indicators of endogenous circadian rhythms such as 24-hour cortisol and melatonin, as these measures are unavailable within this dataset. Third, lack of information on shift work schedule could potentially confound our findings. Nevertheless, exclusion of those who reached peak activity during overnight hours (i.e., between 23:00 and 04:00) did not change our results, thus suggesting that the potential impact of shift work on our findings would be minimal. Lastly, data on neurodegenerative diseases, which have been associated with disrupted rest-activity patterns, were not available. Therefore, future studies are needed to confirm our results and to explore potential mechanisms underlying the association between obesity and RAR.

## Conclusion

Aberrant RAR, as objectively derived from accelerometry data, is significantly and independently related with greater odds of both general and abdominal obesity. These associations are evident across multiple indices of excess adiposity, are similar manifest in older and younger adults but appear to be stronger in women. Longitudinal studies are needed to confirm our findings and to determine whether disrupted RAR can predict future weight gain and obesity.

## Data Availability Statement

The datasets presented in this study can be found in online repositories. The names of the repository/repositories and accession number(s) can be found below: https://wwwn.cdc.gov/nchs/nhanes/continuousnhanes/default.aspx?BeginYear=2011.

## Ethics Statement

The NHANES protocols involving human participants were reviewed and approved by National Center for Health Statistics (NCHS) Ethics Review Board. The patients/participants provided their written informed consent to participate in this study.

## Author Contributions

NC, VKS, FL-J, and JL contributed to the study proposal development. JD and JL contributed to data abstraction and data analysis. NC, JL, JD, VKS, and FL-J contributed to the interpretation of study results. JL, NC, and SV contributed the manuscript drafting. All authors critically reviewed and edited the manuscript before submission.

## Funding

JL is supported by grant 82004301 from the National Natural Science Foundation of China. VKS is supported in part by grants HL65176, HL134885 and HL134808 from National Institutes of Health the Alice Sheets Marriott Professorship, and by funding from the Sleep Number Corporation to Mayo Clinic. NC is supported by grants HL134885 and HL134808 from National Institutes of Health, Mayo Clinic Marie Ingalls Research Career Development Award and a grant from Sleep Number Corporation to Mayo Clinic. The funding bodies had no roles in the design of the study; the collection, analysis, and interpretation of data; the writing of the manuscript; or the decision to submit the manuscript for publication.

## Conflict of Interest

VKS has served as a consultant for Respicardia, Baker Tilly, Bayer and Jazz Pharmaceuticals and serves on the Sleep Number Research Advisory Board.

The remaining authors declare that the research was conducted in the absence of any commercial or financial relationships that could be construed as a potential conflict of interest.

## Publisher’s Note

All claims expressed in this article are solely those of the authors and do not necessarily represent those of their affiliated organizations, or those of the publisher, the editors and the reviewers. Any product that may be evaluated in this article, or claim that may be made by its manufacturer, is not guaranteed or endorsed by the publisher.

## Abbreviations

BMI, body mass index; CI, confidence interval; MIMS, Monitor-Independent Movement Summary units; NHANES, National Health and Nutrition Examination Survey; IS, interdaily stability; IV, intradaily variability; OR, odds ratio; RAR, rest-activity rhythm; SAD, sagittal abdominal diameter; SD, standard deviation; WhtR, waist-to-height ratio.

## References

[1] Pischon T Boeing H Hoffmann K Bergmann M Schulze MB Overvad K. General and Abdominal Adiposity and Risk of Death in Europe. New Engl J Med (2008) 359(20):2105–20. doi: 10.1056/NEJMoa0801891 19005195

[2] Prospective Studies C Whitlock G Lewington S Sherliker P Clarke R Emberson J. Body-Mass Index and Cause-Specific Mortality in 900 000 Adults: Collaborative Analyses of 57 Prospective Studies. Lancet (2009) 373(9669):1083–96. doi: 10.1016/S0140-6736(09)60318-4 PMC2662372 19299006

[3] Kolotkin RL Meter K Williams GR. Quality of Life and Obesity. Obes Rev (2001) 2(4):219–29. doi: 10.1046/j.1467-789x.2001.00040.x 12119993

[4] NCD Risk Factor Collaboration (NCD-RisC). Worldwide Trends in Body-Mass Index, Underweight, Overweight, and Obesity From 1975 to 2016: A Pooled Analysis of 2416 Population-Based Measurement Studies in 128.9 Million Children, Adolescents, and Adults. Lancet (2017) 390(10113):2627–42. doi: 10.1016/S0140-6736(17)32129-3 PMC5735219 29029897

[5] Laermans J Depoortere I. Chronobesity: Role of the Circadian System in the Obesity Epidemic. Obes Rev (2016) 17(2):108–25. doi: 10.1111/obr.12351 26693661

[6] Turek FW Joshu C Kohsaka A Lin E Ivanova G McDearmon E. Obesity and Metabolic Syndrome in Circadian Clock Mutant Mice. Science (2005) 308(5724):1043–5. doi: 10.1126/science.1108750 PMC3764501 15845877

[7] Bandin C Martinez-Nicolas A Ordovas JM Ros Lucas JA Castell P Silvente T. Differences in Circadian Rhythmicity in Clock 3111t/C Genetic Variants in Moderate Obese Women as Assessed by Thermometry, Actimetry and Body Position. Int J Obes (Lond) (2013) 37(8):1044–50. doi: 10.1038/ijo.2012.180 PMC4426980 23183326

[8] Garaulet M Lee YC Shen J Parnell LD Arnett DK Tsai MY. Genetic Variants in Human Clock Associate With Total Energy Intake and Cytokine Sleep Factors in Overweight Subjects (GOLDN Population). Europ J Hum Genet (2010) 18(3):364–9. doi: 10.1038/ejhg.2009.176 PMC2987209 19888304

[9] Sookoian S Gemma C Gianotti TF Burgueno A Castano G Pirola CJ. Genetic Variants of Clock Transcription Factor Are Associated With Individual Susceptibility to Obesity. Am J Clin Nutr (2008) 87(6):1606–15. doi: 10.1093/ajcn/87.6.1606 18541547

[10] Scheer FA Hilton MF Mantzoros CS Shea SA. Adverse Metabolic and Cardiovascular Consequences of Circadian Misalignment. Proc Natl Acad Sci U S A (2009) 106(11):4453–8. doi: 10.1073/pnas.0808180106 PMC2657421 19255424

[11] Sun M Feng W Wang F Li P Li Z Li M. Meta-Analysis on Shift Work and Risks of Specific Obesity Types. Obes Rev (2018) 19(1):28–40. doi: 10.1111/obr.12621 28975706

[12] Antunes LC Levandovski R Dantas G Caumo W Hidalgo MP. Obesity and Shift Work: Chronobiological Aspects. Nutr Res Rev (2010) 23(1):155–68. doi: 10.1017/S0954422410000016 20122305

[13] Yang S Liu A Weidenhammer A Cooksey RC McClain D Kim MK. The Role of Mper2 Clock Gene in Glucocorticoid and Feeding Rhythms. Endocrinology (2009) 150(5):2153–60. doi: 10.1210/en.2008-0705 PMC2671901 19179447

[14] Smith MT McCrae CS Cheung J Martin JL Harrod CG Heald JL. Use of Actigraphy for the Evaluation of Sleep Disorders and Circadian Rhythm Sleep-Wake Disorders: An American Academy of Sleep Medicine Clinical Practice Guideline. J Clin Sleep Med (2018) 14(7):1231–7. doi: 10.5664/jcsm.7230 PMC6040807 29991437

[15] Leng Y Musiek ES Hu K Cappuccio FP Yaffe K. Association Between Circadian Rhythms and Neurodegenerative Diseases. Lancet Neurol (2019) 18(3):307–18. doi: 10.1016/s1474-4422(18)30461-7 PMC6426656 30784558

[16] Musiek ES Bhimasani M Zangrilli MA Morris JC Holtzman DM Ju YS. Circadian Rest-Activity Pattern Changes in Aging and Preclinical Alzheimer Disease. JAMA Neurol (2018) 75(5):582–90. doi: 10.1001/jamaneurol.2017.4719 PMC5885197 29379963

[17] Li P Gao L Gaba A Yu L Cui L Fan W. Circadian Disturbances in Alzheimer’s Disease Progression: A Prospective Observational Cohort Study of Community-Based Older Adults. Lancet Healthy Longev (2020) 1(3):e96–105. doi: 10.1016/s2666-7568(20)30015-5 34179863 PMC8232345

[18] Hoopes EK Witman MA D’Agata MN Berube FR Brewer B Malone SK. Rest-Activity Rhythms in Emerging Adults: Implications for Cardiometabolic Health. Chronobiol Int (2021) 38(4):543–56. doi: 10.1080/07420528.2020.1868490 PMC8035288 33435741

[19] Zuurbier LA Luik AI Hofman A Franco OH Van Someren EJ Tiemeier H. Fragmentation and Stability of Circadian Activity Rhythms Predict Mortality: The Rotterdam Study. Am J Epidemiol (2015) 181(1):54–63. doi: 10.1093/aje/kwu245 25491893

[20] Garaulet M Martinez-Nicolas A Ruiz JR Konstabel K Labayen I Gonzalez-Gross M. Fragmentation of Daily Rhythms Associates With Obesity and Cardiorespiratory Fitness in Adolescents: The Helena Study. Clin Nutr (2017) 36(6):1558–66. doi: 10.1016/j.clnu.2016.09.026 27890490

[21] Quante M Cespedes Feliciano EM Rifas-Shiman SL Mariani S Kaplan ER Rueschman M. Association of Daily Rest-Activity Patterns With Adiposity and Cardiometabolic Risk Measures in Teens. J Adolesc Health (2019) 65(2):224–31. doi: 10.1016/j.jadohealth.2019.02.008 PMC6650322 31056236

[22] Qian J Martinez-Lozano N Tvarijonaviciute A Rios R Scheer F Garaulet M. Blunted Rest-Activity Rhythms Link to Higher Body Mass Index and Inflammatory Markers in Children. Sleep (2021) 44(5):zsaa256. doi: 10.1093/sleep/zsaa256 33249510 PMC8120335

[23] Sohail S Yu L Bennett DA Buchman AS Lim AS. Irregular 24-Hour Activity Rhythms and the Metabolic Syndrome in Older Adults. Chronobiol Int (2015) 32(6):802–13. doi: 10.3109/07420528.2015.1041597 PMC4542004 26061588

[24] Despres JP. Body Fat Distribution and Risk of Cardiovascular Disease: An Update. Circulation (2012) 126(10):1301–13. doi: 10.1161/CIRCULATIONAHA.111.067264 22949540

[25] Merilahti J Viramo P Korhonen I. Wearable Monitoring of Physical Functioning and Disability Changes, Circadian Rhythms and Sleep Patterns in Nursing Home Residents. IEEE J BioMed Health Inform (2016) 20(3):856–64. doi: 10.1109/JBHI.2015.2420680 25861091

[26] National Health and Nutrition Examination Survey-2011-2012 Data Documentation, Codebook, and Frequencies. Available at: https://wwwn.cdc.gov/nchs/nhanes/2011-2012/paxmin_g.htm (Accessed Feb. 18, 2021).

[27] Berkemeyer K Wijndaele K White T Cooper AJM Luben R Westgate K. The Descriptive Epidemiology of Accelerometer-Measured Physical Activity in Older Adults. Int J Behav Nutr Phys Act (2016) 13(1):2. doi: 10.1186/s12966-015-0316-z 26739758 PMC4704380

[28] Li J Somers VK Lopez-Jimenez F Di J Covassin N. Demographic Characteristics Associated With Circadian Rest-Activity Rhythm Patterns: A Cross-Sectional Study. Int J Behav Nutr Phys Act (2021) 18(1):107. doi: 10.1186/s12966-021-01174-z 34407852 PMC8371768

[29] John D Tang Q Albinali F Intille S. An Open-Source Monitor-Independent Movement Summary for Accelerometer Data Processing. J Meas Phys Behav (2019) 2(4):268–81. doi: 10.1123/jmpb.2018-0068 PMC8301210 34308270

[30] Xiao Q Qian J Evans DS Redline S Lane NE Ancoli-Israel S. Cross-Sectional and Prospective Associations of Rest-Activity Rhythms With Metabolic Markers and Type 2 Diabetes in Older Men. Diabetes Care (2020) 43(11):2702–12. doi: 10.2337/dc20-0557 PMC7576417 32887712

[31] Leng Y Blackwell T Cawthon PM Ancoli-Israel S Stone KL Yaffe K. Association of Circadian Abnormalities in Older Adults With an Increased Risk of Developing Parkinson Disease. JAMA Neurol (2020) 77(10):1270–8. doi: 10.1001/jamaneurol.2020.1623 PMC7296450 32539075

[32] Marler MR Gehrman P Martin JL Ancoli-Israel S. The Sigmoidally Transformed Cosine Curve: A Mathematical Model for Circadian Rhythms With Symmetric Non-Sinusoidal Shapes. Stat Med (2006) 25(22):3893–904. doi: 10.1002/sim.2466 16381069

[33] Van Someren EJ Swaab DF Colenda CC Cohen W McCall WV Rosenquist PB. Bright Light Therapy: Improved Sensitivity to Its Effects on Rest-Activity Rhythms in Alzheimer Patients by Application of Nonparametric Methods. Chronobiol Int (1999) 16(4):505–18. doi: 10.3109/07420529908998724 10442243

[34] Cornier MA Despres JP Davis N Grossniklaus DA Klein S Lamarche B. Assessing Adiposity: A Scientific Statement From the American Heart Association. Circulation (2011) 124(18):1996–2019. doi: 10.1161/CIR.0b013e318233bc6a 21947291

[35] Kroenke K Spitzer RL Williams JB. The Phq-9: Validity of a Brief Depression Severity Measure. J Gen Intern Med (2001) 16(9):606–13. doi: 10.1046/j.1525-1497.2001.016009606.x PMC1495268 11556941

[36] Cleland CL Hunter RF Kee F Cupples ME Sallis JF Tully MA. Validity of the Global Physical Activity Questionnaire (GPAQ) in Assessing Levels and Change in Moderate-Vigorous Physical Activity and Sedentary Behaviour. BMC Public Health (2014) 14:1255. doi: 10.1186/1471-2458-14-1255 25492375 PMC4295403

[37] Rao JNK Scott AJ. On Chi-Squared Tests for Multiway Contingency Tables With Cell Proportions Estimated From Survey Data. Ann Stat (1984) 12(1):46–60. doi: 10.1214/aos/1176346391

[38] Cespedes Feliciano EM Quante M Weng J Mitchell JA James P Marinac CR. Actigraphy-Derived Daily Rest-Activity Patterns and Body Mass Index in Community-Dwelling Adults. Sleep (2017) 40(12):zsx168. doi: 10.1093/sleep/zsx168 PMC5804986 29029250

[39] Connor Gorber S Tremblay M Moher D Gorber B. A Comparison of Direct Vs. Self-Report Measures for Assessing Height, Weight and Body Mass Index: A Systematic Review. Obes Rev (2007) 8(4):307–26. doi: 10.1111/j.1467-789X.2007.00347.x 17578381

[40] Mule A Bruno E Pasanisi P Galasso L Castelli L Caumo A. Sex Differences in Rest-Activity Circadian Rhythm in Patients With Metabolic Syndrome. Front Physiol (2021) 12:641461. doi: 10.3389/fphys.2021.641461 33815145 PMC8013705

[41] Smith U. Abdominal Obesity: A Marker of Ectopic Fat Accumulation. J Clin Invest (2015) 125(5):1790–2. doi: 10.1172/JCI81507 PMC4463217 25932676

[42] Decoda Study G Nyamdorj R Qiao Q Lam TH Tuomilehto J Ho SY. BMI Compared With Central Obesity Indicators in Relation to Diabetes and Hypertension in Asians. Obes (Silver Spring) (2008) 16(7):1622–35. doi: 10.1038/oby.2008.73 18421260

[43] Ashwell M Gunn P Gibson S. Waist-To-Height Ratio Is a Better Screening Tool Than Waist Circumference and BMI for Adult Cardiometabolic Risk Factors: Systematic Review and Meta-Analysis. Obes Rev (2012) 13(3):275–86. doi: 10.1111/j.1467-789X.2011.00952.x 22106927

[44] Powell-Wiley TM Poirier P Burke LE Despres JP Gordon-Larsen P Lavie CJ. Obesity and Cardiovascular Disease: A Scientific Statement From the American Heart Association. Circulation (2021) 143(21):e984–1010. doi: 10.1161/CIR.0000000000000973 33882682 PMC8493650

[45] Micklesfield LK Goedecke JH Punyanitya M Wilson KE Kelly TL. Dual-Energy X-Ray Performs as Well as Clinical Computed Tomography for the Measurement of Visceral Fat. Obesity (2012) 20(5):1109–14. doi: 10.1038/oby.2011.367 PMC3343346 22240726

[46] Froy O Garaulet M. The Circadian Clock in White and Brown Adipose Tissue: Mechanistic, Endocrine, and Clinical Aspects. Endocr Rev (2018) 39(3):261–73. doi: 10.1210/er.2017-00193 PMC6456924 29490014

[47] Li Y Ma J Yao K Su W Tan B Wu X. Circadian Rhythms and Obesity: Timekeeping Governs Lipid Metabolism. J Pineal Res (2020) 69(3):e12682. doi: 10.1111/jpi.12682 32656907

[48] Gifkins J Johnston A Loudoun R. The Impact of Shift Work on Eating Patterns and Self-Care Strategies Utilised by Experienced and Inexperienced Nurses. Chronobiol Int (2018) 35(6):811–20. doi: 10.1080/07420528.2018.1466790 29737884

[49] St-Onge MP Ard J Baskin ML Chiuve SE Johnson HM Kris-Etherton P. Meal Timing and Frequency: Implications for Cardiovascular Disease Prevention: A Scientific Statement From the American Heart Association. Circulation (2017) 135(9):e96–121. doi: 10.1161/CIR.0000000000000476 28137935 PMC8532518

[50] McHill AW Phillips AJ Czeisler CA Keating L Yee K Barger LK. Later Circadian Timing of Food Intake Is Associated With Increased Body Fat. Am J Clin Nutr (2017) 106(5):1213–9. doi: 10.3945/ajcn.117.161588 PMC5657289 28877894

[51] McHill AW Melanson EL Higgins J Connick E Moehlman TM Stothard ER. Impact of Circadian Misalignment on Energy Metabolism During Simulated Nightshift Work. Proc Natl Acad Sci USA (2014) 111(48):17302–7. doi: 10.1073/pnas.1412021111 PMC4260578 25404342

[52] Allison KC Hopkins CM Ruggieri M Spaeth AM Ahima RS Zhang Z. Prolonged, Controlled Daytime Versus Delayed Eating Impacts Weight and Metabolism. Curr Biol (2021) 31(4):908. doi: 10.1016/j.cub.2021.01.077 33621495 PMC7971800

[53] Lewis P Oster H Korf HW Foster RG Erren TC. Food as a Circadian Time Cue - Evidence From Human Studies. Nat Rev Endocrinol (2020) 16(4):213–23. doi: 10.1038/s41574-020-0318-z 32055029

[54] Wang Y Kuang Z Yu X Ruhn KA Kubo M Hooper LV. The Intestinal Microbiota Regulates Body Composition Through NFIL3 and the Circadian Clock. Science (2017) 357(6354):912–6. doi: 10.1126/science.aan0677 PMC5702268 28860383

[55] Voigt RM Forsyth CB Green SJ Mutlu E Engen P Vitaterna MH. Circadian Disorganization Alters Intestinal Microbiota. PLoS One (2014) 9(5):e97500. doi: 10.1371/journal.pone.0097500 24848969 PMC4029760

[56] Guo Y Rong Y Huang X Lai H Luo X Zhang Z. Shift Work and the Relationship With Metabolic Syndrome in Chinese Aged Workers. PLoS One (2015) 10(3):e0120632. doi: 10.1371/journal.pone.0120632 25761114 PMC4356508

[57] Qian J Morris CJ Caputo R Wang W Garaulet M Scheer F. Sex Differences in the Circadian Misalignment Effects on Energy Regulation. Proc Natl Acad Sci U S A (2019) 116(47):23806–12. doi: 10.1073/pnas.1914003116 PMC6876189 31685618

[58] Willis EA Creasy SA Honas JJ Melanson EL Donnelly JE. The Effects of Exercise Session Timing on Weight Loss and Components of Energy Balance: Midwest Exercise Trial 2. Int J Obes (Lond) (2020) 44(1):114–24. doi: 10.1038/s41366-019-0409-x PMC6925313 31289334

[59] Kaneko K Yamada T Tsukita S Takahashi K Ishigaki Y Oka Y. Obesity Alters Circadian Expressions of Molecular Clock Genes in the Brainstem. Brain Res (2009) 1263:58–68. doi: 10.1016/j.brainres.2008.12.071 19401184

