# Supplementary material for: Rest-Activity Rhythm Is Associated With Obesity Phenotypes: A Cross-Sectional Analysis
Source: Front Endocrinol (Lausanne). 2022 Jun 28;13:907360. doi: 10.3389/fendo.2022.907360 (PMC9273840; doi:10.3389/fendo.2022.907360)
Supplement: Supplementary file 1 [file DataSheet_1.docx]

**Supplementary files**

| **Contents** | **Page number** |
| --- | --- |
|  |  |
| Supplementary Table 1. Characteristics of the non-pregnant US adult population, the included population, and the excluded population from NHANES 2011-2014 | 2 |
| Supplementary Table 2. Definition of rest-activity rhythm measures | 3 |
| Supplementary Table 3. Detailed methods used for anthropometric measures | 4 |
| Supplementary Table 4. Details of covariates | 5 |
| Supplementary Table 5. Association between standardized continuous RAR metrics (Z-scores) and obesity phenotypes | 6 |
| Supplementary Table 6. R-squared for the linear regression model of standardized continuous RAR metrics (Z-scores) with obesity measures | 7 |
| Supplementary Table 7. Sensitivity analysis results by additional adjustment for objective and self-reported physical activity, depression, sleep disorder and analgesic use | 8-9 |
| Supplementary Table 8. Sensitivity analysis results of the association of Pseudo-F statistic with abdominal obesity stratified by sex | 10 |
| Supplementary Table 9. Mean (SE) of IS and IV in the subset of participants with full 7-day data (n=6005). | 11 |
| Supplementary Table 10. Multivariable linear regression results of the association between IS and IV and obesity measures in the subset of participants with full 7-day data (n=6005). | 12 |
| Supplementary Figure 1. Study flow chart | 13 |
| Supplementary Figure 2. Distribution of rest-activity rhythm measures in the studied sample | 14 |
| Supplementary Figure 3. Scatter plots for bivariate correlations between each pair of RAR measures | 15 |
| Supplementary Figure 4. Distribution of amounts of missing time (minutes) among person-days with non-wear time | 16 |
| Supplementary Figure 5. Stratified analysis of the association of RAR and general obesity by sex and age | 17 |
| Supplementary Figure 6. Stratified analysis of the association of RAR and abdominal obesity by sex and age | 18 |
| Supplementary Figure 7. Sensitivity analysis of the association of RAR parameters with obesity phenotypes by excluding participants reaching their peak activity between 23:00 to 04:00 (n=7790) | 19 |
| Supplementary Figure 8. Sensitivity analysis of the association of RAR parameters with obesity phenotypes by excluding person-days with non-wear time (n=7829) | 20 |

**Supplementary Table 1. Characteristics of the non-pregnant US adult population, the included population, and the excluded population from NHANES 2011-2014**

| **Characteristics** | **All non-pregnant adults**  **(N=9399)** | **Included population**  **(n=7838)** | **Excluded population**  **(n=1561)** |
| --- | --- | --- | --- |
| Men, n (%) | 4559 (48.0) | 3830 (48.1) | 729 (47.4) |
| Age group, years, n (%) | | | |
| 20-39 | 3054 (35.3) | 2464 (32.5) | 590 (53.9) |
| 40-59 | 3108 (38.5) | 2728 (39.4) | 380 (32.5) |
| ≥60 | 2834 (26.2) | 2646 (28.1) | 188 (13.6) |
| Race, n (%) | | | |
| Hispanic | 1912 (14.4) | 1695 (14.4) | 217 (14.0) |
| NH-White | 3646 (66.9) | 3197 (67.2) | 449 (64.3) |
| NH-Black | 2103 (11.2) | 1820 (10.9) | 283 (12.7) |
| NH-Asian | 1071 (4.9) | 896 (4.6) | 175 (6.5) |
| Married/living with a partner, n (%) | 5421 (62.4) | 4570 (63.1) | 851 (58.5) |
| Ever attended college, n (%) | 5067 (63.2) | 4393 (63.2) | 674 (63.3) |
| Employed | 4999 (62.0) | 4245 (60.7) | 754 (70.7) |
| Income poverty ratio <1, n (%) | 1938 (16.1) | 1680 (15.7) | 258 (18.4) |
| Smoking status, n (%) | | | |
| Never | 5098 (56.0) | 4421 (55.7) | 891 (57.2) |
| Former | 2065 (24.0) | 1859 (24.9) | 324 (20.3) |
| Current | 1826 (20.0) | 1551 (19.5) | 345 (22.5) |
| Alcohol consumption, n (%) | 6320 (78.5) | 5366 (78.6) | 954 (78.4) |
| Diet quality, n (%) | | | |
| Excellent/very good | 2840 (32.0) | 2363 (32.5) | 477 (29.8) |
| Good | 4016 (42.4) | 3364 (42.6) | 652 (41.8) |
| Fair/poor | 2538 (25.5) | 2107 (25.0) | 431 (28.3) |
| Health status, n (%) | | | |
| Excellent/very good | 3162 (42.5) | 2698 (43.0) | 464 (39.6) |
| Good | 3513 (39.5) | 2982 (39.5) | 531 (39.8) |
| Fair/poor | 2048 (18.0) | 1713 (17.5) | 335 (20.6) |
| Sleep duration, hrs, mean (SE) | 6.91 (0.02) | 6.91 (0.02) | 6.88 (0.03) |

All estimates accounted for complex survey design. Data are presented as number with weighted percentage (%) or weighted means with standard error (SE). NH, non-Hispanic; SE, standard error.

**Supplementary Table 2. Definition of rest-activity rhythm measures.**

| **RAR measures** | **Definitions** |
| --- | --- |
| Amplitude | Difference between the peak and trough of the fitted extended cosinor curve, representing the magnitude or height of the RAR, with a higher value indicating stronger RAR (i.e., higher activity during the day and lower activity during the night). |
| Mesor | Calculated as value of the function minimum (i.e., trough of the extended cosinor curve, corresponding to activity during rest phase) plus ½ of amplitude, it indicates the mean activity level of the fitted curve. It represents the rhythm-adjusted mean activity level, with higher values indicating higher average activity levels. |
| Acrophase | Clock time to reach peak activity level of the fitted curve, and an indicator of RAR phase. Earlier or later acrophase suggests advanced or delayed RAR, respectively. |
| Pseudo-F statistic | An adjusted measure of goodness-of-fit accounting for the number of observations and parameters, reflects how well the data fit extended cosinor model. It represents robustness of the rhythm with higher values indicating greater rhythmicity. |
| Interdaily stability | Index of day-to-day RAR stability, ranging from 0 to 1, with higher values indicating greater stability and consistency across days. |
| Intradaily variability | Measure of RAR fragmentation across the 24 hours, it ranges from 0 to 2, with higher values representing more fragmented rhythms (i.e., frequent transitions between rest and activity intervals). |

**Supplementary Table 3. Detailed methods used for anthropometric measures**

| **Measures** | **Methods used** |
| --- | --- |
| Height (cm) | Obtained in standing position using a stadiometer |
| Weight (kg) | Measured using a digital weight scale |
| Waist circumference (cm) | Measured at the level of uppermost lateral border of the iliac crest |
| Sagittal abdominal diameter (SAD, cm) | Measured using an abdominal caliper with the participants lying down, and defined as the distance between the small of the back at the iliac crest level and the front of the abdomen, |

**Supplementary Table 4. Details of covariates**

| Education | Categorized into 2 categories: Ever attended college or not |
| --- | --- |
| Employment status | Determined by the question ‘Which of the following were you doing last week?’. Those who responded ‘working at a job or business’ or ‘with a job or business but not at work’ were regarded as employed, while those who answered ‘looking for a job’ or ‘not working at a job or business’ were considered as unemployed. |
| Marital status | Recategorized into married /living with a partner (‘Married’ or ‘living with a partner’) or not (‘Widowed’, ‘divorced’, ‘separated’ or ‘never married’) |
| Poverty income ratio | Coded as a binary variable (< 1 or ≥1) |
| Diet quality | Recoded into 3 g categories: Excellent/very good, good, or fair/poor |
| Self-reported general health status | Recoded into 3 categories: Excellent/very good, good, or fair/poor |
| Alcohol consumption | Had ≥12 alcohol drinks per year or not. |
| Smoking status | Recoded into 3 categories: never, former, current. |
| Depression | Defined as a score ≥ 10 at the Patient Health Questionnaire-9 (PHQ-9). |
| Analgesic use | Self-reported any use of analgesics including opioids (Narcotic analgesics or narcotic analgesic combinations) or nonopioid analgesics (miscellaneous analgesics, nonsteroidal anti-inflammatory agents, salicylates, analgesic combinations, antimigraine agents, COX-2 inhibitors or CGRP inhibitors) in the past 30 days (Code: Multum Lexicon Classification: 57 central nervous system agents [level 1], 58 analgesics [level 2]) |
| Diagnosed sleep disorders | Defined by the question ‘Have you ever been told by a doctor or other health professional that you have a sleep disorder?’. |
| Sleep duration | Determined by the question: “How much sleep (hours) do you usually get at night on weekdays or workdays?” |

**Supplementary Table 5. Association between standardized continuous RAR metrics (Z-scores) and obesity phenotypes**

| **RAR measures (Z-scores)** | **General obesity** | | | **Abdominal obesity** | | |
| --- | --- | --- | --- | --- | --- | --- |
|  | β | OR (95% CI) | *P* | β | OR (95% CI) | *P* |
| Amplitude | **-0.35** | 0.71  (0.64-0.79) | <.001 | **-0.28** | 0.76  (0.68-0.85) | <.001 |
| Mesor | **-0.33** | 0.72  (0.65-0.80) | <.001 | **-0.26** | 0.77  (0.68-0.87) | <.001 |
| Acrophase | 0.01 | 1.01  (0.93-1.09) | .82 | 0.02 | 1.02  (0.93-1.12) | .68 |
| Pseudo-F statistic -women | **-0.12** | 0.88  (0.83-0.94) | <.001 | **-0.27** | 0.76  (0.67-0.86) | <.001 |
| Pseudo-F statistic -men |  |  |  | -0.09 | 0.92  (0.84-1.002) | 0.056 |
| IS | **-0.25** | 0.78  (0.72-0.83) | <.001 | **-0.19** | 0.83  (0.75-0.91) | <.001 |
| IV | **0.42** | 1.53  (1.40-1.66) | <.001 | **0.40** | 1.48  (1.37-1.61) | <.001 |

Adjusted variables included age, sex, race, education, marital status, employment status, poverty income ratio, diet quality, self-reported general health status, alcohol consumption, smoking status and sleep duration and. CI, confidence interval; IS, interdaily stability; IV, intradaily variability; OR, odds ratio.

**Supplementary Table 6. R-squared for the linear regression model of standardized continuous RAR metrics (Z-scores) with obesity measures**

| **RAR measures**  **(Z-score)** | **BMI**  **(n=7838)** | **Waist circumference (n=7838)** | **WHtR**  **(n=7838)** | **SAD**  **(n=7667)** | **Total body fat percentage (n=4382)** | **Trunk fat percentage (n=4567)** |
| --- | --- | --- | --- | --- | --- | --- |
| Amplitude | 0.15 | 0.20 | 0.21 | 0.22 | 0.57 | 0.43 |
| Mesor | 0.14 | 0.19 | 0.21 | 0.22 | 0.57 | 0.43 |
| Acrophase | 0.13 | 0.18 | 0.20 | 0.21 | 0.56 | 0.42 |
| Pseudo-F statistic | 0.14 | 0.19 | 0.20 | 0.21 | 0.56 | 0.43 |
| IS | 0.15 | 0.20 | 0.21 | 0.22 | 0.57 | 0.43 |
| IV | 0.17 | 0.23 | 0.24 | 0.25 | 0.58 | 0.45 |

Adjusted variables included age, sex, race, education, marital status, employment status, poverty income ratio, diet quality, self-reported general health status, alcohol consumption, smoking status, and sleep duration. BMI, body mass index; IS, interdaily stability; IV, intradaily variability; SAD, sagittal abdominal diameter; WHtR, waist-to-height ratio.

**Supplementary Table 7. Sensitivity analysis results by additional adjustment forobjective and self-reported physical activity, depression, sleep disorder and analgesic use.**

| **RAR measures** | **Additional adjustment for mesor** | **Additional adjustment for self-reported PA** | **Additional adjustment for depression** | **Additional adjustment for diagnose sleep disorders** | **Additional adjustment for analgesic use** |
| --- | --- | --- | --- | --- | --- |
| **General obesity** | | | | | |
| Amplitude |  |  |  |  |  |
| Q1 | 1.48 (1.05 -2.08) | 2.35 (1.75-3.17) | 2.27 (1.75-2.96) | 2.16 (1.65 -2.82) | 2.28 (1.76-2.96) |
| Q2 | 1.27 (0.99 -1.61) | 1.78 (1.43-2.22) | 1.78 (1.48 -2.15) | 1.64 (1.33 -2.03) | 1.76 (1.46 -2.12) |
| Q3 | 1.16 (0.92 -1.47) | 1.44 (1.16-1.80) | 1.40 (1.16-1.70) | 1.45 (1.16 -1.81) | 1.40 (1.16 -1.70) |
| Q4 | reference | reference | reference | reference | reference |
| Mesor |  |  |  |  |  |
| Q1 | **-** | 2.49 (1.82-3.40) | 2.39 (1.86 -3.08) | 1.98 (1.51 -2.60) | 2.36 (1.84 -3.04) |
| Q2 | **-** | 1.80 (1.53-2.12) | 1.78 (1.53 -2.08) | 1.60 (1.36 -1.88) | 1.75 (1.50 -2.03) |
| Q3 | **-** | 1.34 (1.03-1.75) | 1.32(1.02 -1.71) | 1.14 (0.92 -1.40) | 1.31 (1.01 -1.70) |
| Q4 | **-** | reference | reference | reference | reference |
| Acrophase |  |  |  |  |  |
| Advanced (<12:44) | 1.33 (0.99-1.77) | 1.06 (0.76-1.47) | 1.03 (0.80 -1.32) | 0.99 (0.69 -1.41) | 1.03 (0.80 -1.33) |
| Delayed (≥16:51) | 1.23 (0.95 -1.61) | 1.14 (0.88-1.47) | 1.13 (0.88 -1.46) | 1.21 (0.96 -1.51) | 1.14 (0.88 -1.48) |
| Normal (12:44-<16:51) | reference | reference | reference | reference | reference |
| Pseudo-F statistic |  |  |  |  |  |
| Q1 | 1.35 (1.12 -1.92) | 1.26 (1.01-1.56) | 1.34 (1.10 -1.62) | 1.52 (1.22 -1.89) | 1.33 (1.10 -1.60) |
| Q2 | 1.30 (1.11 -1.52) | 1.25 (1.01-1.55) | 1.35 (1.15 -1.58) | 1.61 (1.29 -2.02) | 1.36 (1.16 -1.59) |
| Q3 | 1.47 (1.21 -1.79) | 1.45 (1.13-1.86) | 1.53 (1.26 -1.86) | 1.34 (1.05 -1.72) | 1.53 (1.26 -1.87) |
| Q4 | reference | reference | reference | reference | reference |
| IS |  |  |  |  |  |
| Q1 | 1.54 (1.23 -1.93) | 1.83 (1.37-2.45) | 1.89 (1.52 -2.35) | 1.48 (1.12 -1.95) | 1.87 (1.51 -2.33) |
| Q2 | 1.44 (1.15 -1.80) | 1.69 (1.30-2.20) | 1.71 (1.36 -2.14) | 1.43 (1.10 -1.88) | 1.67 (1.33 -2.09) |
| Q3 | 1.18 (0.95 -1.47) | 1.37 (1.04-1.80) | 1.34 (1.07 -1.68) | 1.28 (0.99 -1.65) | 1.33 (1.07 -1.65) |
| Q4 | reference | reference | reference | reference | reference |
| IV |  |  |  |  |  |
| Q1 | reference | reference | reference | reference | reference |
| Q2 | 1.23 (0.96 -1.57) | 1.29 (0.98-1.70) | 1.32 (1.04 -1.67) | 1.40 (1.05 -1.86) | 1.31 (1.05 -1.65) |
| Q3 | 1.70 (1.38 -2.10) | 1.91 (1.50-2.44) | 1.89 (1.54 -2.32) | 2.01 (1.58-2.55) | 1.88 (1.54 -2.30) |
| Q4 | 2.47 (1.87 -3.26) | 2.91 (2.11-4.00) | 2.83 (2.20 -3.66) | 2.45 (1.88-3.20) | 2.80 (2.18 -3.60) |
| **Abdominal obesity** | | | | | |
| Amplitude |  |  |  |  |  |
| Q1 | 1.62 (1.19 -2.15) | 2.32 (1.73-3.12) | 2.17 (1.69 -2.80) | 2.17 (1.69 -2.80) | 2.24 (1.70 -2.93) |
| Q2 | 1.33 (1.05 -1.70) | 1.64 (1.30-2.07) | 1.70 (1.41 -2.05) | 1.70 (1.41 -2.05) | 1.69 (1.36 -2.08) |
| Q3 | 1.31 (1.02-1.59) | 1.42 (1.08 -1.85) | 1.39 (1.15 -1.68) | 1.39 (1.15 -1.68) | 1.45 (1.16 -1.82) |
| Q4 | reference | reference | reference | reference | reference |
| Mesor |  |  |  |  |  |
| Q1 | **-** | 2.23 (1.64 -3.02) | 2.24 (1.74-2.88) | 2.24 (1.74 -2.88) | 2.07 (1.59-2.70) |
| Q2 | **-** | 1.62 (1.34 -1.95) | 1.70 (1.46 -1.98) | 1.70 (1.46 -1.98) | 1.62 (1.38 -1.91) |
| Q3 | **-** | 1.15 (0.92-1.44) | 1.29 (0.99 -1.68) | 1.29 (0.99 -1.68) | 1.15 (0.93 -1.42) |
| Q4 | **-** | reference | reference | reference | reference |
| Acrophase |  |  |  |  |  |
| Advanced (<12:44) | 1.22 (0.84 -1.78) | 1.10 (0.71 -1.72) | 1.05 (0.81 -1.37) | 1.05 (0.81 -1.37) | 0.97 (0.69 -1.37) |
| Delayed (≥16:51) | 1.30 (1.03 -1.64) | 1.29 (1.02 -1.64) | 1.15 (0.89- 1.48) | 1.15 (0.89 -1.48) | 1.21 (0.96 -1.52) |
| Normal  (12:44-<16:51) | reference | reference | reference | reference | reference |
| Pseudo-F statistic |  |  |  |  |  |
| Women |  |  |  |  |  |
| Q1 | 1.81 (1.30 -2.50) | 1.84 (1.33-2.54) | 1.79 (1.30 -2.47) | 1.79 (1.31 -2.44) | 3.25 (1.42-7.45) |
| Q2 | 2.02 (1.48 -2.77) | 2.13 (1.53-2.96) | 2.11 (1.52 -2.94) | 2.10 (1.49 -2.95) | 5.19 (2.05-13.15) |
| Q3 | 1.87 (1.42 -2.45) | 1.94 (1.46-2.59) | 1.94 (1.45 -2.60) | 1.92 (1.43 -2.57) | 2.36 (1.10 -5.06) |
| Q4 | reference | reference | reference | reference | reference |
| Men |  |  |  |  |  |
| Q1 | 1.18 (0.91 -1.53) | 1.18 (0.91-1.55) | 1.17 (0.89 -1.54) | 1.14 (0.88 -1.48) | 1.08 (0.47 -2.48) |
| Q2 | 1.11 (0.85 -1.45) | 1.16 (0.88-1.53) | 1.13 (0.86 -1.49) | 1.14 (0.87 -1.51) | 0.91 (0.39 -2.11) |
| Q3 | 0.83 (0.62 -1.10) | 0.86 (0.65-1.16) | 0.85 (0.64 -1.14) | 0.85 (0.64 -1.13) | 0.48 (0.21 -1.10) |
| Q4 | reference | reference | reference | reference | reference |
| IS |  |  |  |  |  |
| Q1 | 1.28 (0.97 -1.68) | 1.39 (1.03 -1.88) | 1.83 (1.48 -2.26) | 1.83 (1.48 -2.26) | 1.51 (1.15 -2.00) |
| Q2 | 1.25 (0.95 -1.66) | 1.51 (1.15 -1.97) | 1.67 (1.35 -2.06) | 1.67 (1.35 -2.06) | 1.43 (1.09 -1.88) |
| Q3 | 1.16 (0.90 -1.50) | 1.17 (0.86 -1.60) | 1.32 (1.05 -1.64) | 1.32 (1.05 -1.64) | 1.28 (0.99 -1.66) |
| Q4 | reference | reference | reference | reference | reference |
| IV |  |  |  |  |  |
| Q1 | reference | reference | reference | reference | reference |
| Q2 | 1.34 (1.03 -1.75) | 1.29 (0.92 -1.80) | 1.28 (1.02 -1.62) | 1.28 (1.02 -1.62) | 1.41 (1.07 -1.87) |
| Q3 | 1.89 (1.52-2.34) | 1.99 (1.53 -2.58) | 1.84 (1.49 -2.26) | 1.84 (1.49 -2.26) | 2.04 (1.62 -2.58) |
| Q4 | 2.27 (1.80 -2.87) | 2.38 (1.72 -3.29) | 2.73 (2.11 -3.53) | 2.73 (2.11- 3.53) | 2.49 (1.92 -3.24) |

Data are presented as odds ratio (95% confidence interval). The highest quartile was designated as reference for amplitude, mesor, pseudo-F statistic, and IS, while the lowest quartile was used as reference for IV.

IS, interdaily stability; IV, intradaily variability; PA, physical activity.

**Supplementary Table 8. Sensitivity analysis results of the association of Pseudo-F statistic with abdominal obesity stratified by sex.**

| **Pseudo-F statistic** | **Exclusion of late acrophase**  **(23:00-04:00) (n=7790)** | | **Exclusion of person-days with non-wear time (n=7829)** | |
| --- | --- | --- | --- | --- |
| Women |  | *P* trend  <.001 |  | *P* trend <.001 |
| Q1 | 1.80 (1.31-2.48) |  | 1.79 (1.32-2.44) |  |
| Q2 | 2.15 (1.52-3.03) |  | 1.99 (1.42-2.79) |  |
| Q3 | 1.95 (1.46-2.61) |  | 1.91 (1.43-2.55) |  |
| Q4 | Reference |  | Reference |  |
| Men |  | *P* trend=  .15 |  | *P* trend=  .10 |
| Q1 | 1.18 (0.90-1.54) |  | 1.03 (0.82-1.30) |  |
| Q2 | 1.12 (0.84-1.50) |  | 1.11 (0.86-1.45) |  |
| Q3 | 0.85 (0.64-1.13) |  | 0.78 (0.62-0.99) |  |
| Q4 | Reference |  | Reference |  |

Data are presented as odds ratio (95% confidence interval).

**Supplementary Table 9. Mean (SE) of IS and IV in the subset of participants with full 7-day data (n=6005)**

| **Characteristics** | **Overall** (n=6005) | **General obesity** | | **Abdominal obesity** | |
| --- | --- | --- | --- | --- | --- |
|  |  | No (n=3684) | Yes (n=2321) | No (n=2596) | Yes (n=3409) |
| IS, mean (SE) | 0.36 (0.002) | 0.37 (0.002) | 0.35 (0.002) | 0.37 (0.003) | 0.36 (0.002) |
| IV, mean (SE) | 0.43 (0.001) | 0.42 (0.001) | 0.45 (0.002) | 0.42 (0.002) | 0.44 (0.002) |

IS, interdaily stability; IV, intradaily variability; SE, standard error.

**Supplementary Table 10. Multivariable linear regression results of the association between IS and IV and obesity measures in the subset of participants with full 7-day data.**

| **RAR measures** | **IS** | | **IV** | |
| --- | --- | --- | --- | --- |
|  | β | *P* value | β | *P* value |
| BMI (n=6005) | **-0.15** | <.001 | **0.21** | <.001 |
| Waist circumference (n=6005) | **-0.15** | <.001 | **0.23** | <.001 |
| WHtR (n=6005) | **-0.14** | <.001 | **0.22** | <.001 |
| SAD (n=5871) | **-0.14** | <.001 | **0.24** | <.001 |
| Total body fat percentage (n=3197) | **-0.09** | <.001 | **0.17** | <.001 |
| Trunk fat percentage (n=3338) | **-0.10** | <.001 | **0.19** | <.001 |

β represents standardized coefficient. Adjusted variables included age, sex, race, education, marital status, employment status, poverty income ratio, diet quality, self-reported general health status, alcohol consumption, smoking status, and sleep duration. BMI, body mass index; IS, interdaily stability; IV, intradaily variability; SAD, sagittal abdominal diameter; WHtR, waist-to-height ratio.


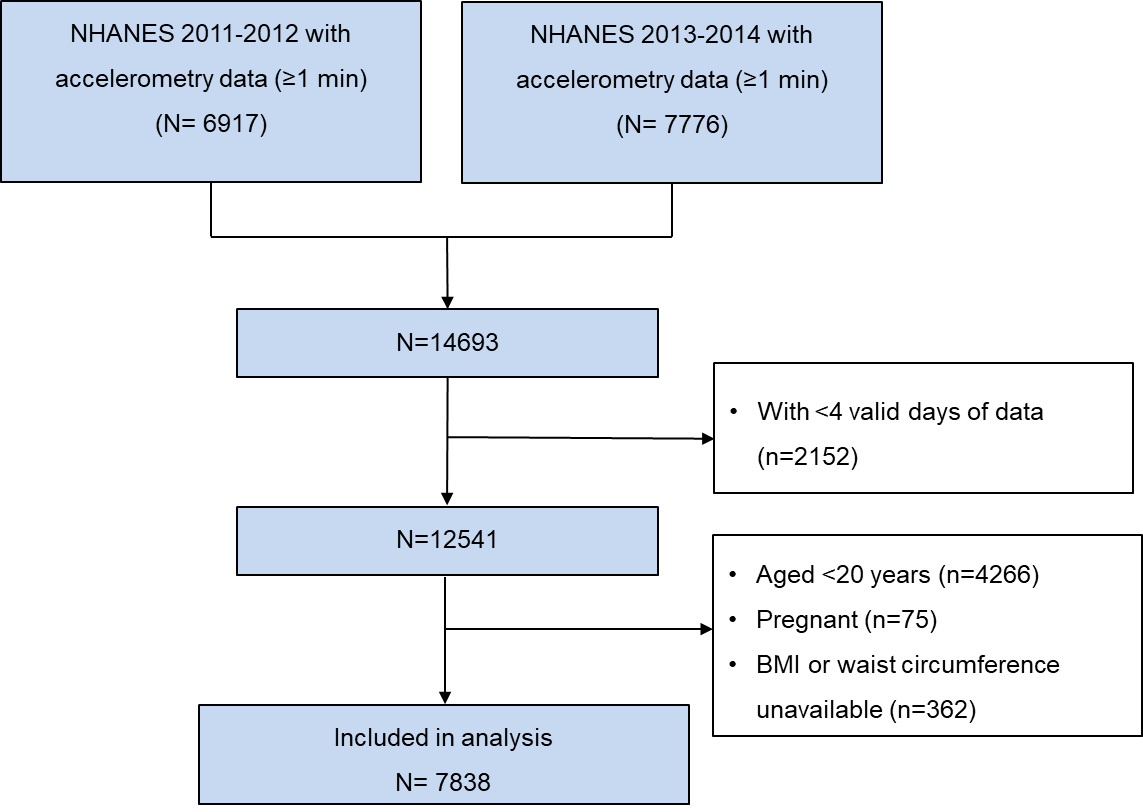


**Supplementary Figure 1. Study flow chart.** NHANES, National Health and Nutrition Examination Survey. BMI, body mass index.


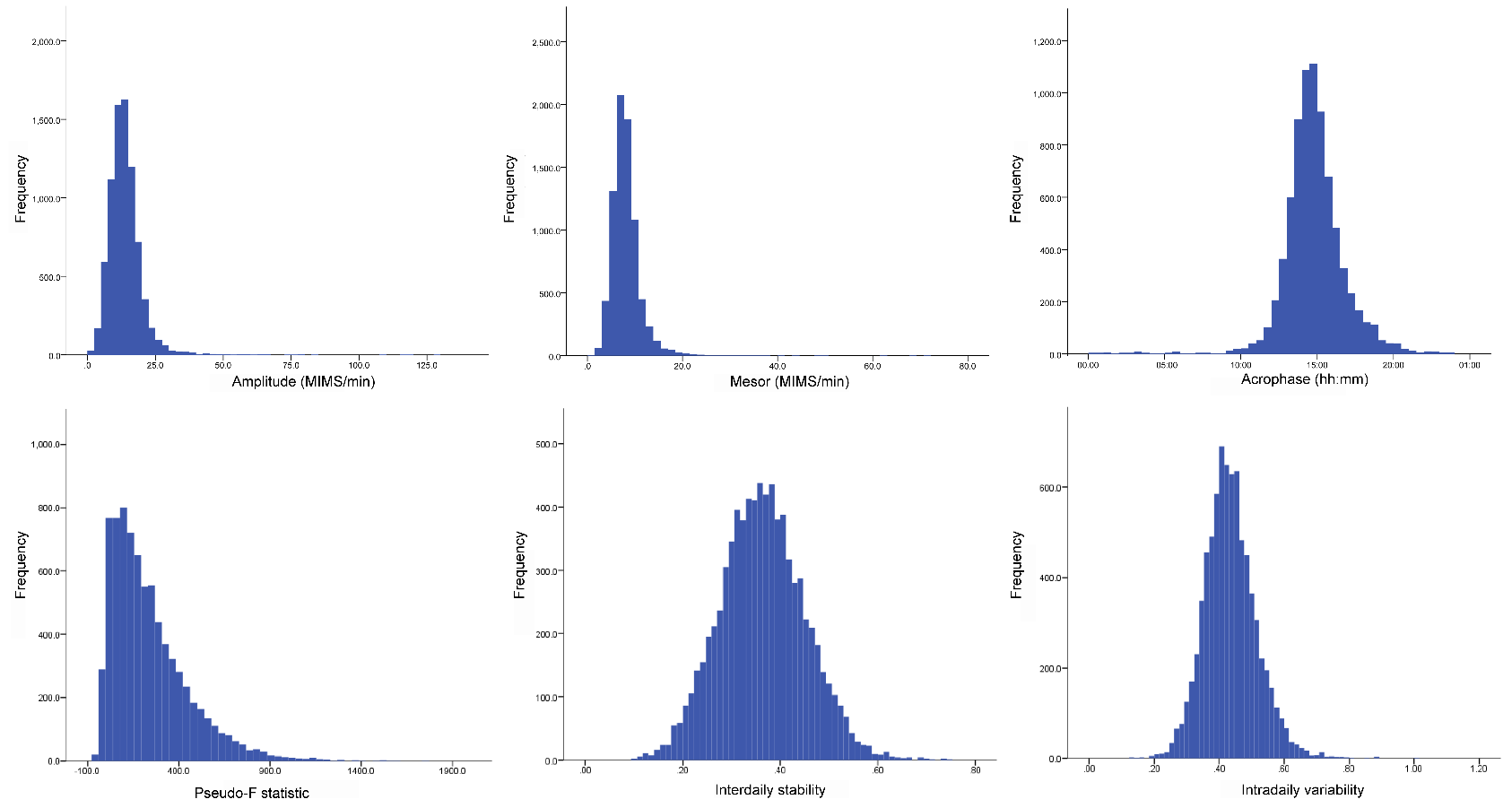


**Supplementary Figure 2. Distribution of rest-activity rhythm measures in the studied sample.**


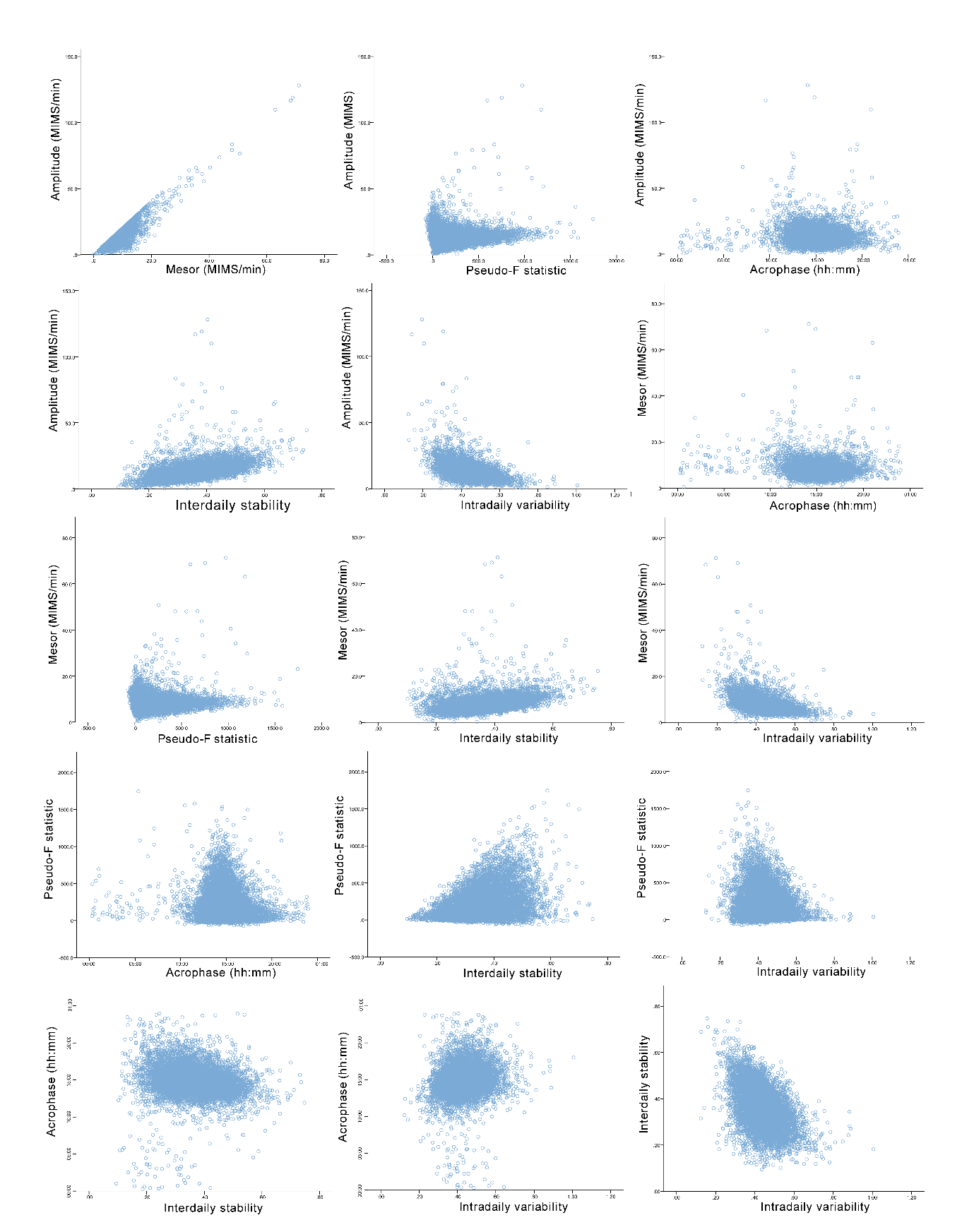


**Supplementary Figure 3. Scatter plots for bivariate correlations between each pair of RAR measures**


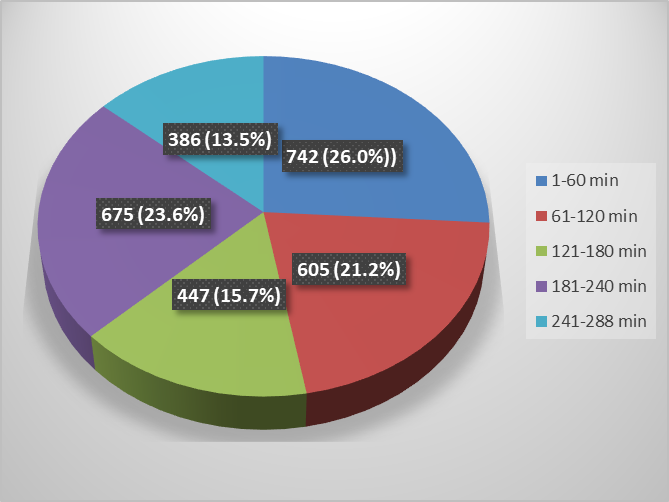


**Supplementary Figure 4. Distribution of amounts of missing time (minutes) among person-days with non-wear time (n=2965 person-days [5.5% of total sample]).** Data are presented as number (%) of person-days.


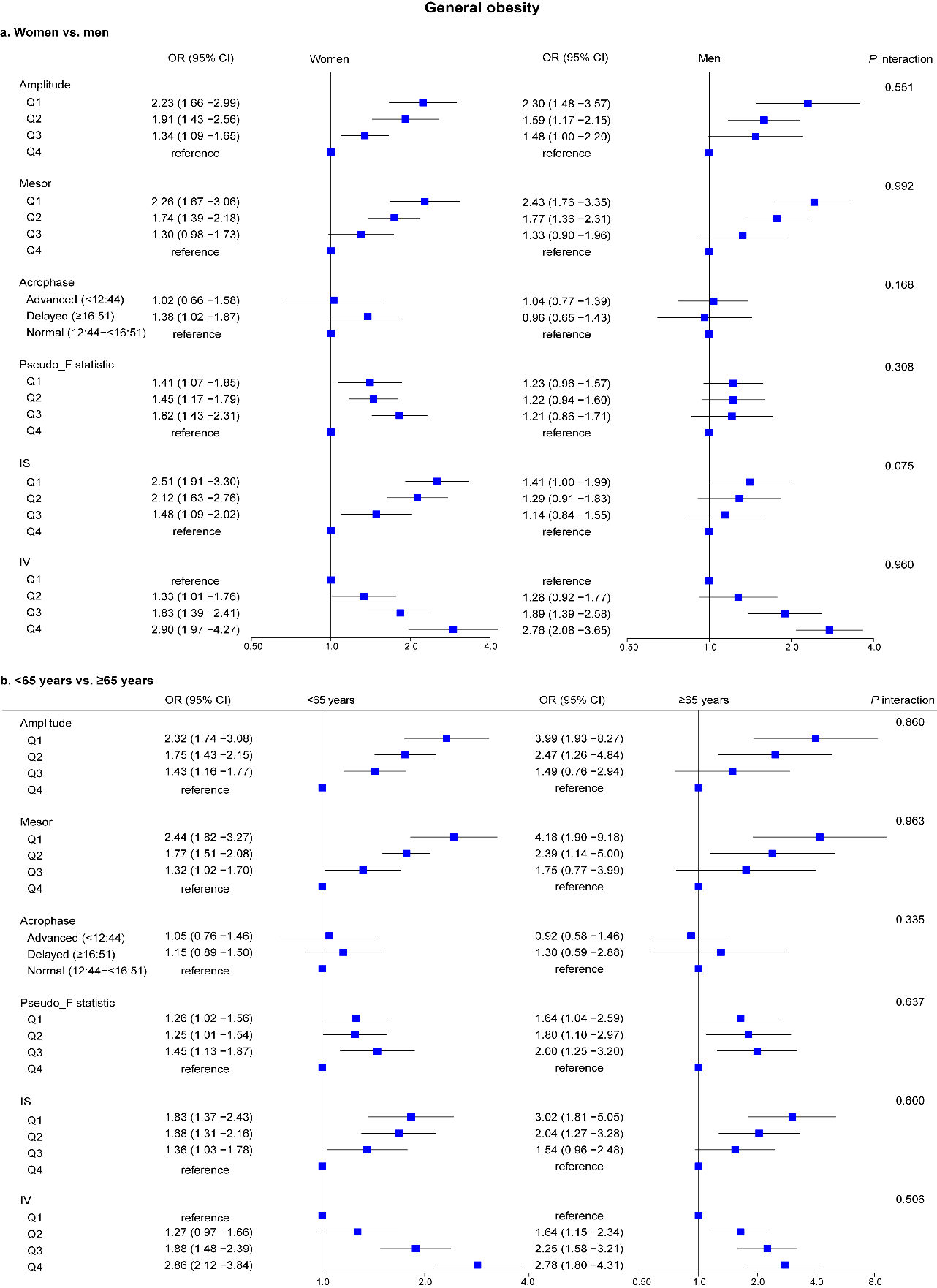


**Supplementary Figure 5. Stratified analysis of the association of RAR and general obesity by sex and age.** Odds ratio (datapoints) and 95% confidence interval (error bars) are presented. The highest quartile was designated as reference for amplitude, mesor, pseudo-F statistic, and IS, while the lowest quartile was used as reference for IV. CI, confidence interval; IS, interdaily stability; IV, intradaily variability; OR, odds ratio.


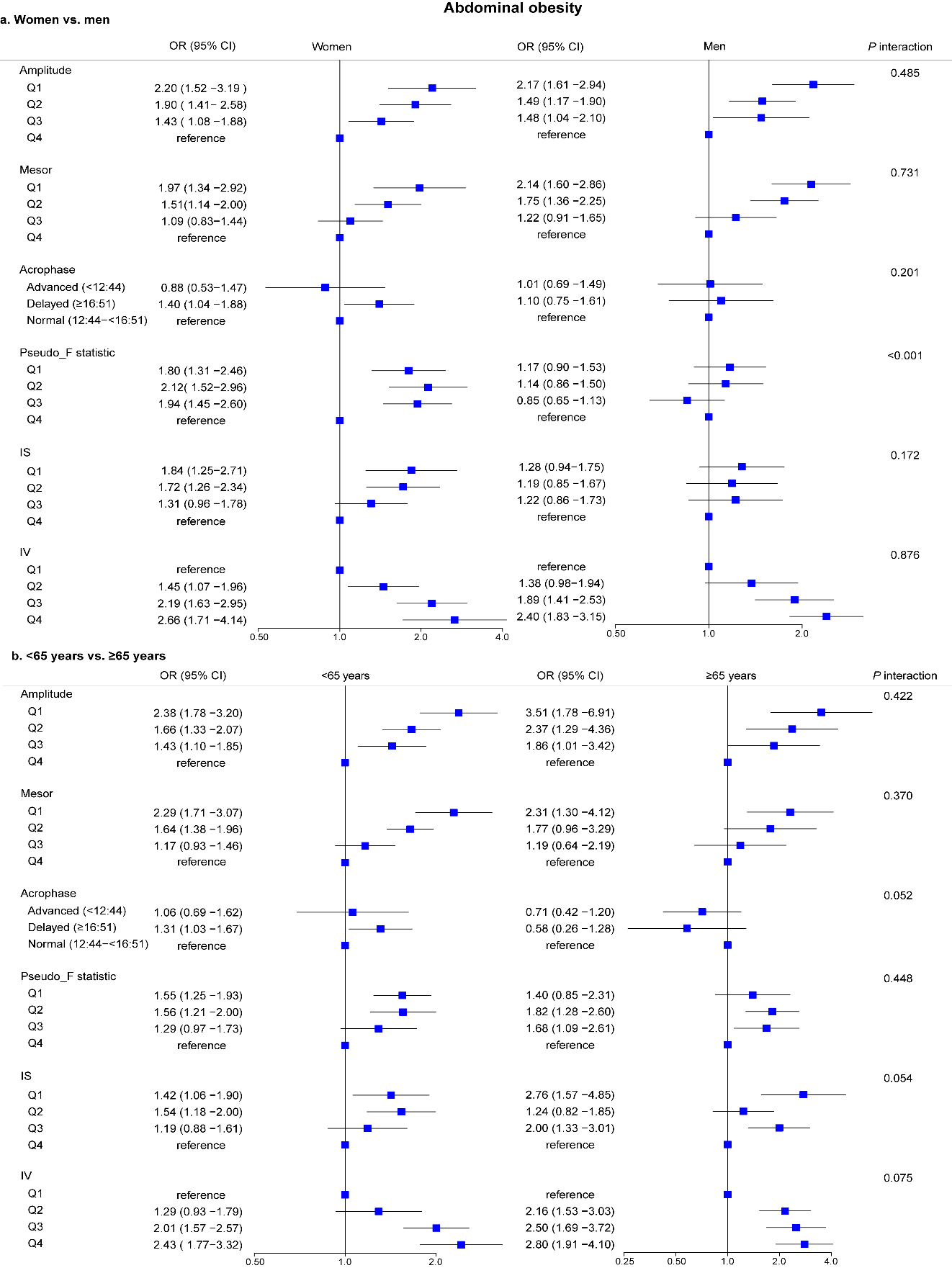


**Supplementary Figure 6. Stratified analysis of the association of RAR and abdominal obesity by sex and age.** Odds ratio (datapoints) and 95% confidence interval (error bars) are presented. The highest quartile was designated as reference for amplitude, mesor, pseudo-F statistic, and IS, while the lowest quartile was used as reference for IV. CI, confidence interval; IS, interdaily stability; IV, intradaily variability; OR, odds ratio.

**
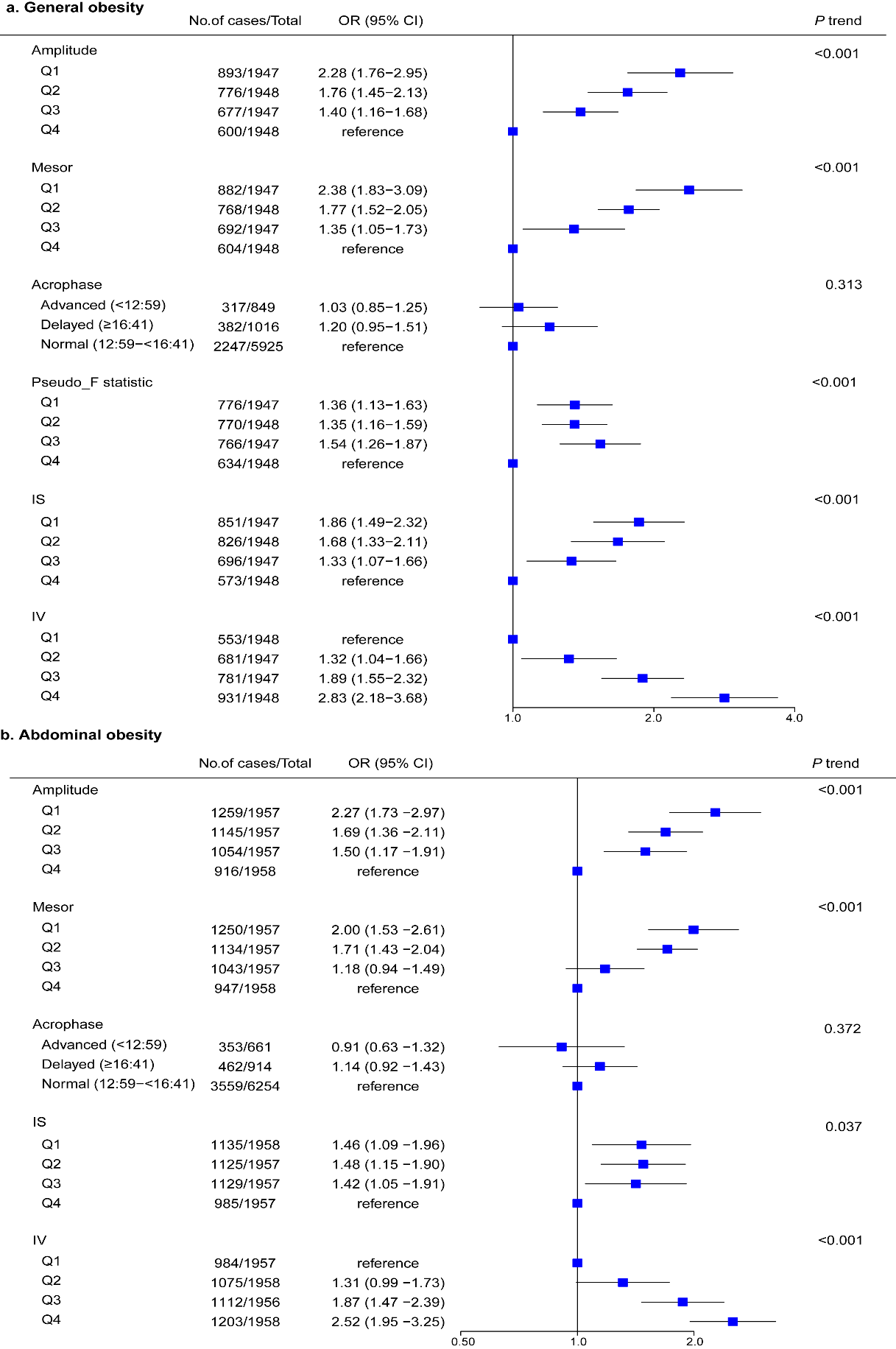
**

**Supplementary Figure 7. Sensitivity analysis of the association of RAR parameters with obesity phenotypes by excluding participants reaching their peak activity between 23:00 and 04:00 (n=7790).** Odds ratio (datapoints) and 95% confidence interval (error bars) are presented. The highest quartile was designated as reference for amplitude, mesor, pseudo-F statistic, and IS, while the lowest quartile was used as reference for IV. CI, confidence interval; IS, interdaily stability; IV, intradaily variability; OR, odds ratio.


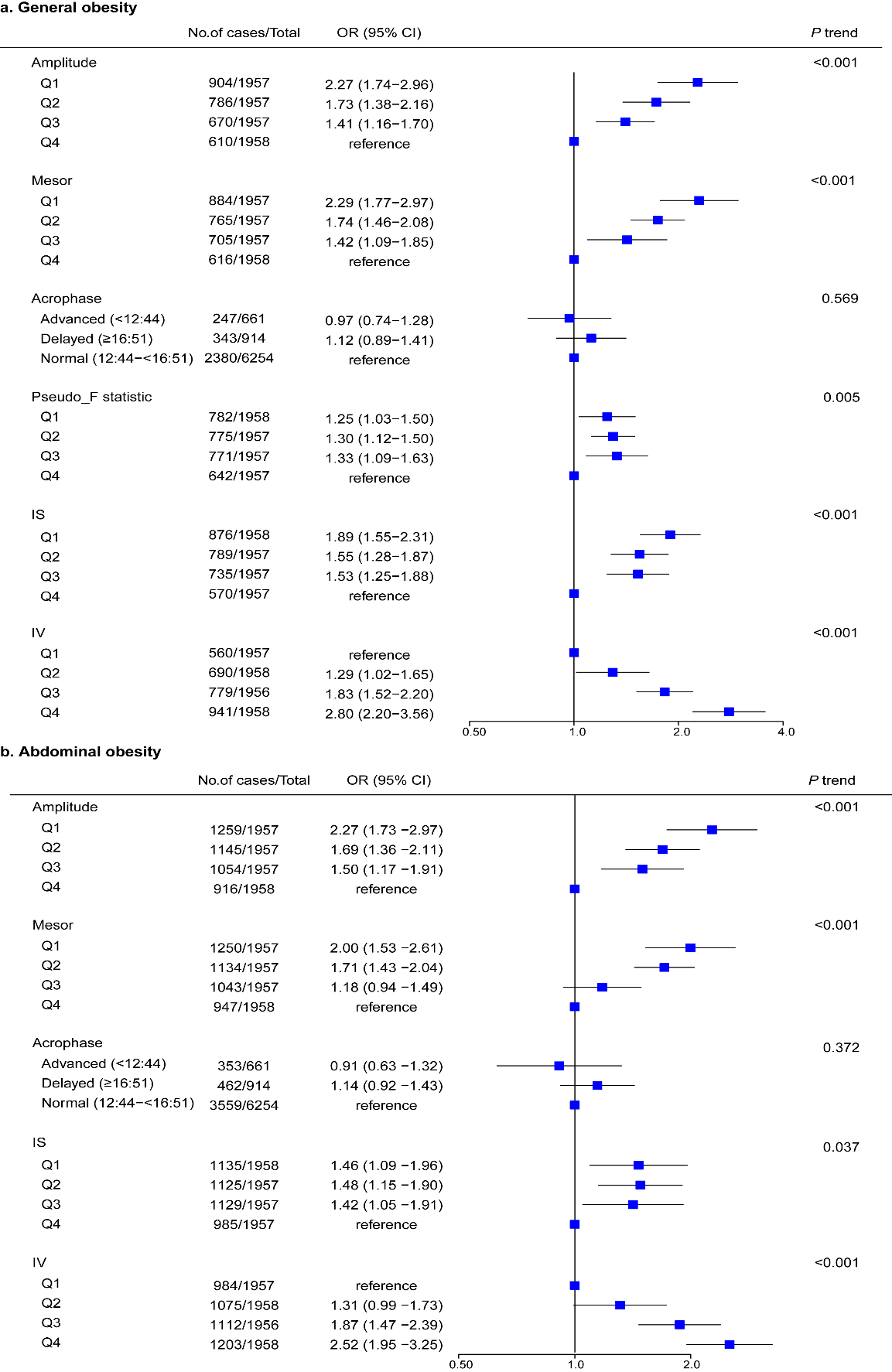


**Supplementary Figure 8, Sensitivity analysis of the association of RAR parameters with obesity phenotypes by excluding person-days with non-wear time (n=7829).** Odds ratio (datapoints) and 95% confidence interval (error bars) are presented. The highest quartile was designated as reference for amplitude, mesor, pseudo-F statistic, and IS, while the lowest quartile was used as reference for IV. CI, confidence interval; IS, interdaily stability; IV, intradaily variability; OR, odds ratio.
